# Ophiostomatalean fungi associated with bark beetles infesting *Pinus armandii* Franch., with characterizations of seven new species from Shaanxi, China

**DOI:** 10.3897/mycokeys.140.199146

**Published:** 2026-09-18

**Authors:** Minjie Chen, Yutong Ran, Congwang Liu, Mingliang Yin

**Affiliations:** 1 College of Forestry and Landscape Architecture, South China Agricultural University, Guangzhou, 510642, China College of Forestry and Landscape Architecture, South China Agricultural University Guangzhou China

**Keywords:** *
Ceratocystiopsis
*, *
Graphilbum
*, *
Leptographium
*, *
Masuyamyces
*, *
Ophiostoma
*, taxonomy

## Abstract

*Pinus armandii* is widely distributed in western China and is of considerable ecological significance. However, recent bark beetle outbreaks have caused severe damage to forests in Shaanxi Province, and these outbreaks are closely associated with ophiostomatalean fungi (*Ascomycota*, *Ophiostomatales*). These fungi not only incite plant diseases but also facilitate the successful establishment of the beetles. At present, knowledge of the ophiostomatalean fungal communities associated with bark beetles infesting *Pinus armandii* in Shaanxi Province remains limited. This study aimed to identify ophiostomatalean fungi associated with *P. armandii* in Ankang City, Shaanxi Province. A total of 230 ophiostomatalean isolates were obtained from 46 gallery samples. Based on multilocus phylogenetic analyses, and morphological and physiological characteristics, we identified 20 species belonging to six genera. Among them were seven novel species, namely *Ceratocystiopsis rugosa***sp. nov**., *Graphilbum prolificum***sp. nov**., *G. ramosus***sp. nov**., *Masuyamyces huoditangensis***sp. nov**., *Ophiostoma flavescens***sp. nov**., *O. lenta***sp. nov**., and *O. mediotumida***sp. nov**., along with two unidentified taxa (*Ceratocystiopsis* sp. 1 and *Leptographium* sp. 1), and eleven previously described species. This study provides a basis for understanding the relationship between the beetles and the ophiostomatalean fungi in Shaanxi Province, China.

## Introduction

Global trade and climate change are facilitating the spread of forest pests into new territories, posing a significant threat to forest health worldwide (Netherer et al. 2024). Bark beetles (*Coleoptera*, *Curculionidae*, *Scolytinae*) are a highly diverse group of insects that commonly infest coniferous trees and are highly concealed (Morris et al. 2017). Some of these species can cause extensive tree mortality (Ferrenberg et al. 2014). In contrast to the impacts of a single harmful organism, the synergistic effects of microbial pathogens and insects infestations tends to result in more serious outcomes for forest ecosystems (Raffa et al. 2008). Fungal symbioses are regarded as key drivers of forest pest outbreaks, among which the associations between bark beetles and ophiostomatalean fungi (*Ascomycota*, *Ophiostomatales*) are particularly prominent, having attracted widespread attention due to the enormous economic losses and ecological damage they cause (Wingfield et al. 2016, 2017; Thakur et al. 2019).

Ophiostomatalean fungi, comprising over 400 species in 20 genera, are the most common fungal associates of bark beetles (De Beer et al. 2022; Wang et al. 2024b, 2024c, 2025). Their characteristic flask-shaped ascomata and sticky spore droplets facilitate their dispersal by bark beetles (Dowding 1984; Malloch and Blackwell 1993). Bark beetles inoculate their fungal partner into trees by carrying spores on their exoskeleton or actively transporting them (Raffa et al. 2015). Additionally, some bark beetles use special structures on their body surface called mycangia or depressions to carry and transmit spores (Batra 1963; Hofstetter et al. 2015; Kostovcik et al. 2015). According to records, these fungi, during the process of colonization, markedly reduce host resistance, promote the successful establishment of bark beetles, supply nutrients essential for the beetles to complete their life cycle, and increase their survival rate (Ayres et al. 2000; Lee et al. 2005; Kirisits 2007; Wadke et al. 2016; Kandasamy et al. 2019). Moreover, many examples demonstrate that ophiostomatalean fungi can produce bark beetle aggregation pheromones, thereby inducing group attacks (Zhao et al. 2015). For example, the bicyclic ketals from *Grosmannia penicillata* (Grosmann) Goid., *Gro. europhioides* (E.F. Wright & Cain) Zipfel, Z.W. de Beer and M.J. Wingf., *Ophiostoma bicolor* R.W. Davidson & D.E. Wells, and *O. piceae* (Münch) Syd. & P. Syd. contain three known semiochemicals (exo-brevicomin, endo-brevicomin, and (5S,7S)-trans-conophthorin), which have been shown to elicit antennal responses in *Ips typographus* (Linnaeus) (Zhao et al. 2019). Ophiostomatalean fungi include species that infect trees or wood, causing sapwood blue-stain or severe tree diseases, as well as species that can cause human diseases (Wingfield et al. 1993; Seifert et al. 2013). Well-known examples include Dutch elm disease (DED) caused by *O. ulmi* (Buisman) Nannf. and *O. novo-ulmi* Brasier (De Hoog 1974; Brasier 1991), black root disease caused by *Leptographium wageneri* (W.B. Kendr.) M.J. Wingf. (Harrington and Cobb 1988), and laurel wilt caused by *Raffaelea lauricola* T.C. Harr., Fraedrich & Aghayeva (Harrington et al. 2008). Consequently, elucidating beetle-fungus associations is critical for studying forest pest outbreaks.

Due to varying tolerances to different environmental factors, such as host tree defensive compounds, water, and oxygen demands, fungal associations may differ temporally or spatially (Solheim 1991; Viiri 1997; Klepzig et al. 2004; Hofstetter et al. 2006; Adams and Six 2007; Six and Bentz 2007; Bleiker and Six 2009a, 2009b). Accordingly, investigations of ophiostomatalean fungi across various regions of China are ongoing and steadily progressing. Multi-locus phylogenetic analyses have facilitated the identification of numerous new species, particularly through investigations of ophiostomatalean fungi associated with *Ips* bark beetles in northeastern, northwestern, and southwestern China, which have led to the description of nearly 60 novel taxa spanning six distinct genera: *Ceratocystiopsis*, *Graphilbum*, *Grosmannia*, *Leptographium*, *Masuyamyces*, and *Ophiostoma* (Liu et al. 2017; Chang et al. 2019; Wang et al. 2020, 2021, 2024b). Recently, six new ophiostomatalean species have been reported in association with *Hylurgus ligniperda* (Fabricius), a bark beetle newly introduced into eastern China (Xie et al. 2025), and two new ophiostomatalean species associated with *Pinus sylvestris* var. *mongolica* Litv. have also been discovered in Heilongjiang (Wang et al. 2025). Collectively, these studies have revealed numerous novel beetle-fungus associations and have facilitated the discovery and description of many new taxa, suggesting that the taxonomic and ecological diversity of ophiostomatalean fungi in China is largely underexplored.

As a native coniferous pioneer tree species in China (Li et al. 2012), *P. armandii* Franch. has been continuously attacked by a variety of bark beetles, primarily *Dendroctonus armandi* Tsai & Li, 1959, resulting in extensive mortality (Tang and Chen 1999). However, research on ophiostomatalean fungi associated with bark beetles infesting *P. armandii* remains limited. To date, only 21 species belonging to five genera have been reported in association with bark beetles attacking *P. armandii* (Hu et al. 2015; Pan et al. 2020; Wang et al. 2022b, 2024b). Among these, *Graphilbum parakesiyea* T. Wang & Q. Lu, *L. qinlingense* (M. Tang) T. Wang & Q. Lu, and *O. shennongense* T. Wang & Q. Lu have been experimentally confirmed to induce lesion formation in the xylem of *P. armandii* (Wang et al. 2024a). Therefore, researching the diversity of ophiostomatalean fungi associated with *P. armandii* is of great scientific significance and practical value for assessing the potential threat of bark beetle-fungus partnerships to *P. armandii* populations.

This study investigated communities of ophiostomatalean fungi associated with bark beetles infesting *P. armandii* in Ankang, Shaanxi, China. Species were identified based on morphological observations, multilocus DNA sequence data, and phylogenetic analyses, following field sampling and laboratory isolation. Our findings provide new insights into the diversity of these fungi and enrich the existing data on ophiostomatalean fungi associated with bark beetles targeting *P. armandii*.

## Materials and methods

### Sample collection and fungal isolation

**Figure 1.**
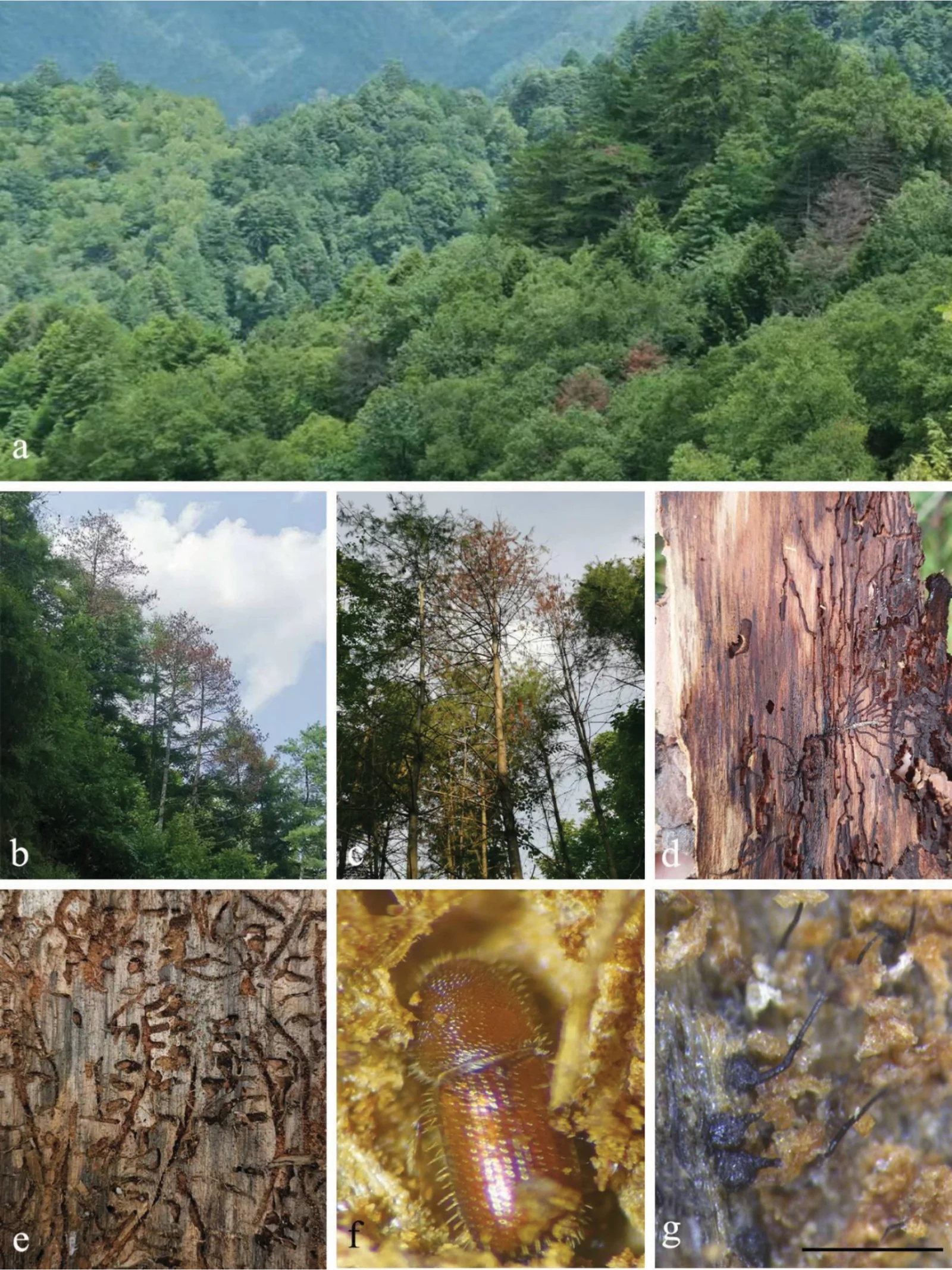
**a–c**. Disease symptoms on *Pinus armandii* infested by bark beetles and ophiostomatalean fungi in Shaanxi, China; **d, e**. Galleries from *P. armandii* infested with bark beetles; **f**. Bark beetles in galleries on *P. armandii*; **g**. Ophiostomatalean fungi in galleries on *P. armandii*. Scale bars: 12 μm (**g**).

In August 2022, 46 bark gallery samples from beetle-infested *P. armandii*, along with several adult beetles, were collected from two sites in Ankang City, Shaanxi Province, China; Huoditang Forest Farm and Chengguan Town (Fig. 1). The collected bark beetles and their galleries were placed into sterile centrifuge tubes and sterile sampling bags, respectively, labelled with numbers, and transported to the laboratory, where they were stored at 4 °C until fungal isolation. The fungi observed in the galleries and pupal chambers were transferred onto 2% malt extract agar plates (90 mm diameter) (MEA: 20 g BioLab malt extract powder, 20 g BioLab agar, and 1,000 ml deionized water) using a stereomicroscope. After inoculation, the plates were incubated in a dark environment at 25 °C for one to two weeks, and then the hyphae at the tip of the colony were cut and inoculated onto MEA plates to obtain pure strains. Following an initial analysis of colony and morphological characteristics, representative strains of each morphotype were selected for further studies. All fungal isolates were preserved at the South China Agricultural University, Guangzhou, Guangdong Province, China (SCAU; for accession numbers, see Table 1). Ex-type cultures of ophiostomatalean fungi described in this study were deposited in the China General Microbiological Culture Collection Center (**CGMCC**), and the holotype specimens (dry cultures) were deposited in the Herbarium Mycologicum, Academiae Sinicae (**HMAS**), Beijing, China.

**Table 1.** Representative isolates of ophiostomatalean fungi associated with bark beetles Infesting *P. armandii* obtained in this study.

**Taxon**	**Species^1^**	**Strain no.^2.3.4^**	**Insect**	**GenBank No.^5^**			
				**βT**	**ITS**	**LSU**	**TEF1-α**
* Ceratocystiopsis *							
1	*Ceratocystiopsis* sp. 1	SCAU 22	*Dendroctonus armandi*	PQ368068	PQ372966	PQ369936	PQ368005
		SN20022					
		SCAU 22	*D. armandi*	PQ368065	PQ372961	PQ369937	PQ368004
		SN22012					
		SCAU 22	*D. armandi*	PQ368063	PQ372964	PQ369938	PQ368009
		SN23113					
		SCAU 22	*D. armandi*	PQ368069	PQ372962	PQ369939	PQ368011
		SN30012					
2	*C. rugosa* sp. nov.	SCAU 22	*Polygraphus* sp.	PQ368059	PQ372969	PQ369944	PQ368012
		SN25011^T^					
		(CGMCC 3.27261)					
		SCAU 22	*Polygraphus* sp.	PQ368056	PQ372972	PQ369945	PQ368013
		SN25031					
		SCAU 22	*Polygraphus* sp.	PQ368058	PQ372973	PQ369946	PQ368016
		SN25111					
		SCAU 22	*Polygraphus* sp.	PQ368061	PQ372974	PQ369947	PQ368014
		SN25221					
		SCAU 22	*Polygraphus* sp.	PQ368060	PQ372970	PQ369949	PQ368017
		SN34141					
		SCAU 22	*Polygraphus* sp.	PQ368057	PQ372971	PQ369948	PQ368015
		SN41141					
3	*C. weihaiensis*	SCAU 22	*Cyrtogenius luteus*	PQ368072	—	—	—
		SN05041					
* Graphilbum *							
4	*G. fragrans*	SCAU 22	*Polygraphus* sp.	PQ381100	—	—	—
		SN15011					
5	*G. parakesiyea*	SCAU 22	*Polygraphus* sp.	PQ381068	—	—	—
		SN05061					
6	*G. prolificum* sp. nov.	SCAU 22	*Polygraphus* sp.	PQ381064	PQ362186	PQ369951	PQ368018
		SN24062					
		SCAU 22	*Polygraphus* sp.	PQ381065	PQ362187	PQ369952	PQ368019
		SN25051^T^					
		(CGMCC					
		3.27262)					
		SCAU 22	*Polygraphus* sp.	PQ381066	PQ362184	PQ369958	PQ368021
		SN31031					
		SCAU 22	*Polygraphus* sp.	PQ381063	PQ362191	PQ369955	PQ368024
		SN33011					
		SCAU 22	*Polygraphus* sp.	PQ381067	PQ362188	PQ369956	PQ368022
		SN41171					
7	*G. ramosus* sp. nov.	SCAU 22	*D. armandi*	PQ381102	PQ362193	PQ369960	PQ368055
		SN42141^T^					
		(CGMCC					
		3.27263)					
		SCAU 22	*D. armandi*	PQ381101	PQ362192	PQ369959	PQ368054
		SN42143					
* Leptographium *							
8	*L. koraiensis*	SCAU 22	*D. armandi*	PQ381074	PQ362200	PQ369962	PQ368033
		SN28061					
		SCAU 22	*D. armandi*	PQ381073	PQ362199	PQ369961	PQ368032
		SN28063					
9	*L. qinlingense*	SCAU 22	*D. armandi*	PQ381070	—	PQ369967	PQ368028
		SN37031					
		SCAU 22	*D. armandi*	PQ381069	—	PQ369966	PQ368029
		SN37033					
10	*Leptographium* sp. 1	SCAU 22	*D. armandi*	PQ381071	PQ362197	PQ369968	PQ368030
		SN45021					
		SCAU 22	*D. armandi*	PQ381072	PQ362198	PQ369969	PQ368031
		SN45101					
GroupA							
11	*Grosmannia taigensis*	SCAU 22	*Polygraphus* sp.	PQ381075	PQ362194	PQ369963	PQ368025
		SN13051					
		SCAU 22	*Polygraphus* sp.	PQ381076	PQ362196	PQ369964	PQ368027
		SN13091					
		SCAU 22	*Polygraphus* sp.	PQ381077	PQ362195	PQ369965	PQ368026
		SN32111					
* Masuyamyces *							
12	*M. huoditangensis* sp. nov.	SCAU 22	*D. armandi*	PQ381079	PQ362203	PQ369971	PQ368047
		SN34132^T^					
		(CGMCC					
		3.27264)					
		SCAU 22	*D. armandi*	PQ381080	PQ362202	PQ369972	PQ368048
		SN40091					
		SCAU 22	*D. armandi*	PQ381081	PQ362201	PQ369970	PQ368046
		SN17232					
		SCAU 22	*D. armandi*	PQ381078	PQ362204	PQ369973	PQ368049
		SN31091					
Group B							
13	*Ophiostoma lenta* sp. nov.	SCAU 22	*Dryocoetes hectographus*	PQ381098	PQ362208	PQ369975	PQ368050
		SN18181 ^T^					
		(CGMCC					
		3.27266)					
		SCAU 22	*D. hectographus*	PQ381099	PQ362207	PQ369974	PQ368051
		SN18183					
14	*O. mediotumida* sp. nov.	SCAU 22	*D. hectographus*	PQ381097	PQ362206	PQ369977	PQ368052
		SN18191 ^T^					
		(CGMCC					
		3.27267)					
		SCAU 22	*D. hectographus*	PQ381096	PQ362205	PQ369976	PQ368053
		SN18193					
* Ophiostoma *							
15	*O. flavescens* sp. nov.	SCAU 22	*D. armandi*	PQ381084	PQ362219	PQ369989	PQ368042
		SN32061^T^					
		(CGMCC					
		3.27265)					
		SCAU 22	*D. armandi*	PQ381085	PQ362220	PQ369990	PQ368043
		SN32113					
16	*O. ips*	SCAU 22	*Cy. luteus*	PQ381083	—	—	—
		SN01021					
17	*O. minus*	SCAU 22	*Cy. luteus*	PQ381095	PQ362210	PQ369979	PQ368034
		SN03282					
18	*O. shennongense*	SCAU 22	*D. armandi*	PQ381092	PQ362218	PQ369987	PQ368044
		SN17231					
		SCAU 22	*D. armandi*	PQ381093	PQ362217	PQ369988	PQ368045
		SN35031					
19	*O. yaluense*	SCAU 22	*D. armandi*	PQ381086	PQ362211	PQ369980	PQ368036
		SN15061					
		SCAU 22	*D. armandi*	PQ381091	PQ362212	PQ369981	PQ368041
		SN15111					
		SCAU 22	*D. armandi*	PQ381089	PQ362213	PQ369982	PQ368039
		SN15132					
		SCAU 22	*D. armandi*	PQ381088	PQ362216	PQ369984	PQ368040
		SN28041					
		SCAU 22	*D. armandi*	PQ381087	PQ362214	PQ369985	PQ368038
		SN33031					
		SCAU 22	*D. armandi*	PQ381090	PQ362215	PQ369986	PQ368037
		SN41031					
* Sporothrix *							
20	*S. pseudoabietina*	SCAU 22	*Polygraphus* sp.	PQ381082	—	—	—
		SN34021					

^1.^ Species named in black bold are novel species in this study. ^2.^SCAU = South China Agricultural University, Guangdong, China ^3.^CGMCC = China General Microbiological Culture Collection ^4. T^ = ex-holotype isolates ^5.^βT = the β-tubulin gene region, ITS = Internal transcribed spacer region of the nuclear ribosomal DNA gene, LSU = Large subunit of the nrDNA gene, TEF1-α = Translation elongation factor 1-alpha.

### DNA extraction and PCR amplification

Before DNA extraction, strains were grown on 2% MEA at 25 °C in the dark for 1–2 weeks. The viable mycelium at the tip of the pure strains were scraped into 1.5 mL sterile centrifuge tubes. According to the instructions, DNA was extracted using PrepMan^TM^ Ultra Sample Preparation Reagent (from Thermo Fisher Scientific).

A total of four DNA regions were amplified for sequencing and phylogenetic analysis. BT2a and BT2b (Glass and Donaldson 1995) primer pairs amplified the βT gene; ITS5 and ITS4 (White et al. 1990) primer pairs amplified the internal transcribed spacer (ITS) regions; the ribosomal large subunit (LSU) region was amplified by LROR and LR5 (Vilgalys and Hester 1990) primer pairs; and EF2F (Marincowitz et al. 2015) and EF2R (Jacobs et al. 2004) primer pairs amplified TEF1-α gene.

PCR detection was performed in 25 μL (12.5 μL 2× Taq Master Mix, 0.5 μL forward and reverse primers, 10.5 μL PCR-grade deionized water, and 1 μL DNA stock solution). The PCR amplification conditions were pre-denaturation at 95 °C for 3 minutes. Denaturation at 95 °C for 30 seconds, annealing at 52**–**58 °C for 30 seconds, extension at 72 °C for 1 minute; a total of 35 cycles; and finally, extension at 72 °C for 10 minutes, followed by holding at 12 °C.

All PCR products were sent to Sangon Biotech Co., Ltd. (Guangzhou, China) for sequencing, and the resulting sequences were downloaded from the company’s website (https://store.sangon.com). The sequences obtained in this study were deposited in GenBank (Table 1) (www.ncbi.nlm.nih.gov/Genbank).

### Phylogenetic analyses

The downloaded sequences were assembled by Geneious v. 7.1.4 (Biomatters, Auckland, New Zealand). The sequences obtained from the four DNA fragments were preliminarily identified by BLAST search in the NCBI GenBank database. The representative sequences with the highest similarity matching and the model strain sequences of similar species were downloaded from the NCBI GenBank database. The GenBank numbers of these sequences are shown on the corresponding phylogenetic trees. The dataset was assembled in MEGA v. 7.0 (Kumar et al. 2016) and compared using the online tool MAFFT v. 7 (Katoh et al. 2019). Phylogenetic analysis was performed using maximum likelihood (ML), maximum parsimony (MP), and Bayesian inference (BI).

The best ML, MP, and BI alternative models were selected in JModelTest 2.1.10 (Darriba et al. 2012). MEGA-X (Kumar et al. 2018) was used for ML analysis with the nearest-neighbor interchange (NNI) branch-swapping option, and node confidence intervals were determined by 1,000 bootstrap replicates. MP analysis was conducted using PAUP v4.0b10 (Swofford 2002), and bootstrap analysis with 1,000 replicates was performed to assess branch-node support. Tree length, consistency index (CI), retention index (RI), rescaled consistency index (RC), and homoplasy index (HI) of the most parsimonious trees were calculated. BI analysis was conducted using MrBayes v3.2.7 (Ronquist and Huelsenbeck 2003). Starting from a random starting tree, four Markov chain Monte Carlo (MCMC) chains were run simultaneously for 5 million generations to calculate posterior probabilities. Trees were sampled every 100 generations, resulting in 50,000 trees. The first 25% of sampled trees were discarded as burn-in, and the posterior probability was calculated from the retained trees. The phylogenetic tree was edited in FigTree v1.4.2.

### Morphological and physiological characteristics

Isolations of potentially undescribed species were transferred onto 2% MEA medium and incubated in the dark at 25 °C. Each isolate was inoculated onto five plates to assess its growth rate. The two orthogonal diameters of the colonies were recorded on the 7^th^ and 14^th^ days of colony growth. Colony colour was assessed using the colour chart of Rayner (1970). All data related to the type specimens were stored in MycoBank.

For microscopic examination, the isolates were inoculated onto 2% water agar (WA, 20 g Difco agar and 1,000 ml deionized water) amended with sterilized pine twigs, and incubated at 25 °C for 3–4 weeks (Duong et al. 2012). After incubation, the sexual or asexual morphs of the isolates were excised and mounted onto microscope slides for microscopic examination. A Zeiss Axio Scope A1 (Carl Zeiss, Germany) was used to measure and photograph the microscopic structures of ophiostomatalean fungi. For the strains selected as holotypes, the lengths and widths of 30 reproductive structures per strain were measured (such as conidia and conidiophores), and the statistics were presented as (min–) (mean – SD)–(mean + SD) (–max) (mean, average; SD, standard deviation; min, minimum; max, maximum).

## Results

### Collection of samples and isolation of fungi

In this study, 230 isolates of ophiostomatoid fungi were isolated from 46 bark gallery samples of *P. armandii*, of which 76 strains were from Chengguan Town and 154 from Huoditang Forest Farm (Table 2). Preliminary identification was colony growth rates and morphological characteristics of these strains. Standard nucleotide BLAST searches against the GenBank database were performed using the βT sequences of all strains for preliminary classification and affinity assessment. Subsequently, 50 representative strains were selected for in-depth morphological study and multilocus phylogenetic analysis.

**Table 2.** Strain numbers and percentages of ophiostomatalean fungi from bark beetle galleries in Shaanxi, China.

**Genus**	**Species**	**Location**	**Number of isolates**	**Percentage (%)**
* Ceratocystiopsis *	*Ceratocystiopsis* sp. 1	Huoditang Forest Farm	21	9.13
	*C. rugosa*	Huoditang Forest Farm	8	3.48
	*C. weihaiensis*	Chengguan Town	16	6.96
* Graphilbum *	*G. fragrans*	Chengguan Town	1	0.43
	*G. parakesiyea*	Chengguan Town	21	9.13
	*G. prolificum*	Huoditang Forest Farm	18	7.83
	*G. ramosus*	Huoditang Forest Farm	2	0.87
* Leptographium *	*L. koraiensis*	Huoditang Forest Farm	2	0.87
	*L. qinlingense*	Huoditang Forest Farm	2	0.87
	*Leptographium* sp. 1	Huoditang Forest Farm	2	0.87
Group A	*Grosmannia taigensis*	Chengguan Town and Huoditang Forest Farm	3	1.30
* Masuyamyces *	*M. huoditangensis*	Huoditang Forest Farm	6	2.61
Group B	*O. lenta*	Huoditang Forest Farm	2	0.87
	*O. mediotumida*	Huoditang Forest Farm	2	0.87
* Ophiostoma *	*O. flavescens*	Huoditang Forest Farm	2	0.87
	*O. ips*	Chengguan Town	26	11.30
	*O. minus*	Chengguan Town	1	0.43
	*O. shennongense*	Huoditang Forest Farm	63	27.39
	*O. yaluense*	Huoditang Forest Farm	11	4.78
* Sporothrix *	*S. pseudoabietina*	Chengguan Town and Huoditang Forest Farm	21	9.13
Total	—	—	230	100.00

### Phylogenetic analysis

Phylograms generated by ML are presented for all datasets, with clade supports indicated by ML, MP, and BI analyses. Initially, phylogenetic analyses were conducted at the genus level based on the βT gene region, followed by a more precise species-level delineation using the ITS, LSU, and TEF1-α gene regions, as well as their concatenated dataset. The phylogenetic analyses revealed that 50 representative strains were distributed across 20 terminal clades. The results are presented in the following order: *Ceratocystiopsis* (Taxa 1, 2), *Graphilbum* (Taxa 6, 7), *Leptographium* and Group A (Taxa 8–11), *Masuyamyces* and Group B (Taxa 12–14), and *Ophiostoma* (Taxon 15, 18, 19). For phylogenetic analyses of each genus, complex, or group, the number of positions and the best nucleotide substitution models (Table 3) of each DNA barcode dataset were used, and the phylogenetic relationships between species are described.

**Table 3.** Datasets information for phylogenetic analysis.

**Genus**	**Other level**	**Dat-asets**	**Positi-ons**	**Nucleotide substitution models**	**Maximum parsimony**				
					**Length**	**CI**	**RI**	**RC**	**HI**
*Ceratocystiopsis*+ *Graphilbum* + *Leptographium* + *Masuyamyces* + *Ophiostoma* + *Sporothrix*	—	βT	470 bp	TrN + I + G	2603	0.385	0.855	0.3	0.615
								2	
								9	
* Ceratocystiopsis *	—	βT	468 bp	GTR + G	1083	0.546	0.876	0.4	0.454
								7	
								8	
		ITS	634 bp	TrN + G	1464	0.624	0.886	0.5	0.376
								5	
								3	
		TEF1-α	625 bp	TrN + G	1167	0.657	0.887	0.5	0.343
								8	
								3	
		βT + ITS + TEF1-α	1727 bp	GTR+I+G	2839	0.667	0.877	0.5	0.333
								8	
								6	
* Graphilbum *	—	βT	640 bp	GTR + I + G	1491	0.649	0.911	0.5	0.351
								9	
								1	
		ITS	592 bp	GTR + I + G	507	0.838	0.964	0.8	0.162
								0	
								8	
		TEF1-α	918 bp	TrN + I + G	2893	0.513	0.864	0.4	0.487
								4	
								3	
*Leptographium* and Group A	—	ITS + LSU	1247 bp	GTR + I + G	1942	0.707	0.951	0.6	0.293
								7	
								3	
	*L. galeiforme* complex	βT + TEF1-α	637 bp	TrN + G	313	0.843	0.881	0.7	0.157
								4	
								3	
	*L. lundbergii* complex	βT + TEF1-α	1069 bp	GTR + G	971	0.694	0.776	0.5	0.306
								3	
								9	
	*L. piceiperdum* complex	βT + TEF1-α	1085 bp	GTR + I	576	0.903	0.940	0.8	0.097
								4	
								8	
	Group A	βT + TEF1-α	1007 bp	GTR + G	451	0.914	0.939	0.8	0.086
								5	
								7	
*Masuyamyces* and Group B	—	βT	576 bp	TrN + I + G	1258	0.671	0.879	0.5	0.329
								8	
								9	
		ITS	539 bp	GTR + G	416	0.752	0.938	0.7	0.248
								0	
								6	
		TEF1-α	694 bp	GTR + I + G	976	0.698	0.893	0.6	0.302
								2	
								3	
* Ophiostoma *	*O. clavatum* complex	βT	345 bp	GTR + I + G	285	0.839	0.959	0.8	0.161
								0	
								4	
		ITS	544 bp	TrN + G	415	0.745	0.944	0.7	0.255
								0	
								3	
		TEF1-α	641 bp	GTR + G	1174	0.716	0.932	0.6	0.284
								6	
								7	
		βT + ITS + TEF1-α	1530 bp	GTR + I + G	2050	0.663	0.890	0.5	0.337
								9	
								0	

(Note: Based on standard nucleotide BLAST searches of the βT sequences against GenBank and phylogenetic analyses of the βT gene region (Fig. 2), six of the taxa were identified as known species: Taxon 3 clustered with *C. weihaiensis* R.L. Chang & X.Y. Zhang (*Ceratocystiopsis*); Taxa 4 and 5 grouped with *G. fragrans* (Math.-Käärik) Z.W. de Beer, Seifert & M.J. Wingf. and *G. parakesiyea* (*Graphilbum*), respectively; Taxa 16 and 17 were placed with *O. ips* (Rumbold) Nannf. and *O. minus* (Hedgc.) Syd. & P. Syd. (*Ophiostoma*), respectively; and Taxon 20 grouped with *S. pseudoabietina* H.M. Wang, Q. Lu & Zhen Zhang (*Sporothrix*). Because these taxa were isolated at high frequencies from bark beetle-associated fungi, we did not sequence additional gene regions or construct phylogenetic inferences using other loci for them. Therefore, these six taxa are not included in the phylogenetic descriptions presented in the following sections.).

**Figure 2.**
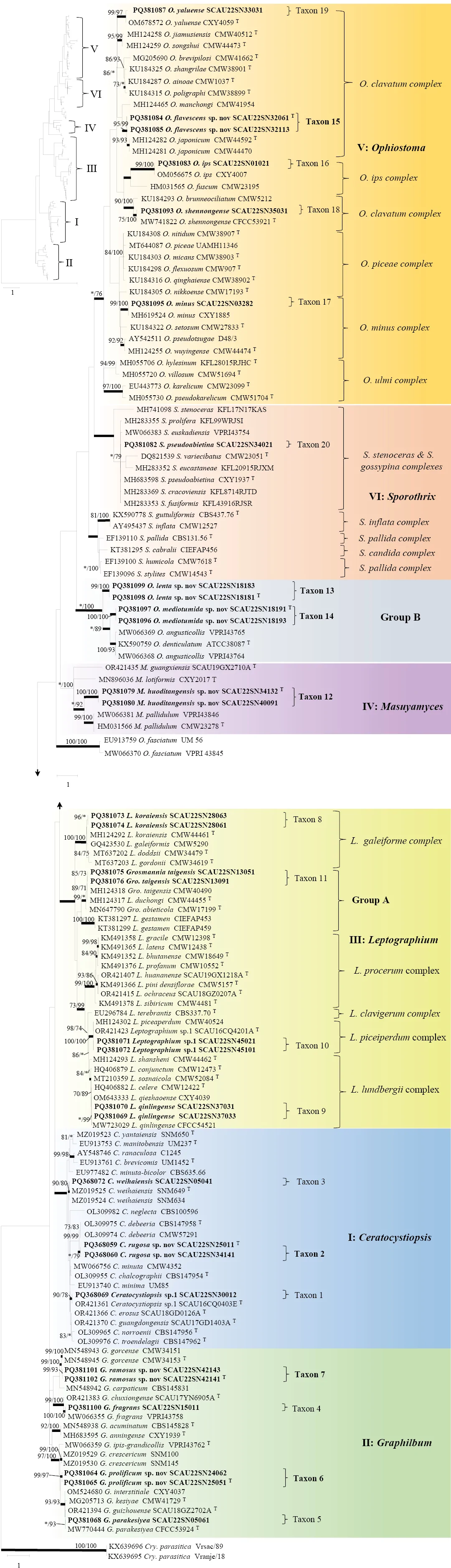
Maximum Likelihood tree of ophiostomatalean fungi (*Ceratocystiopsis* + *Graphilbum* + *Leptographium* + *Masuyamyces* + *Ophiostoma* + *Sporothrix*) from the βT sequence data. Sequences generated from this study are printed in bold. Bold branches indicate posterior probability values ≥ 0.95. Bootstrap values of ML/MP ≥ 70% are recorded at the nodes. T = ex-type isolates.

### Ceratocystiopsis

**Figure 3.**
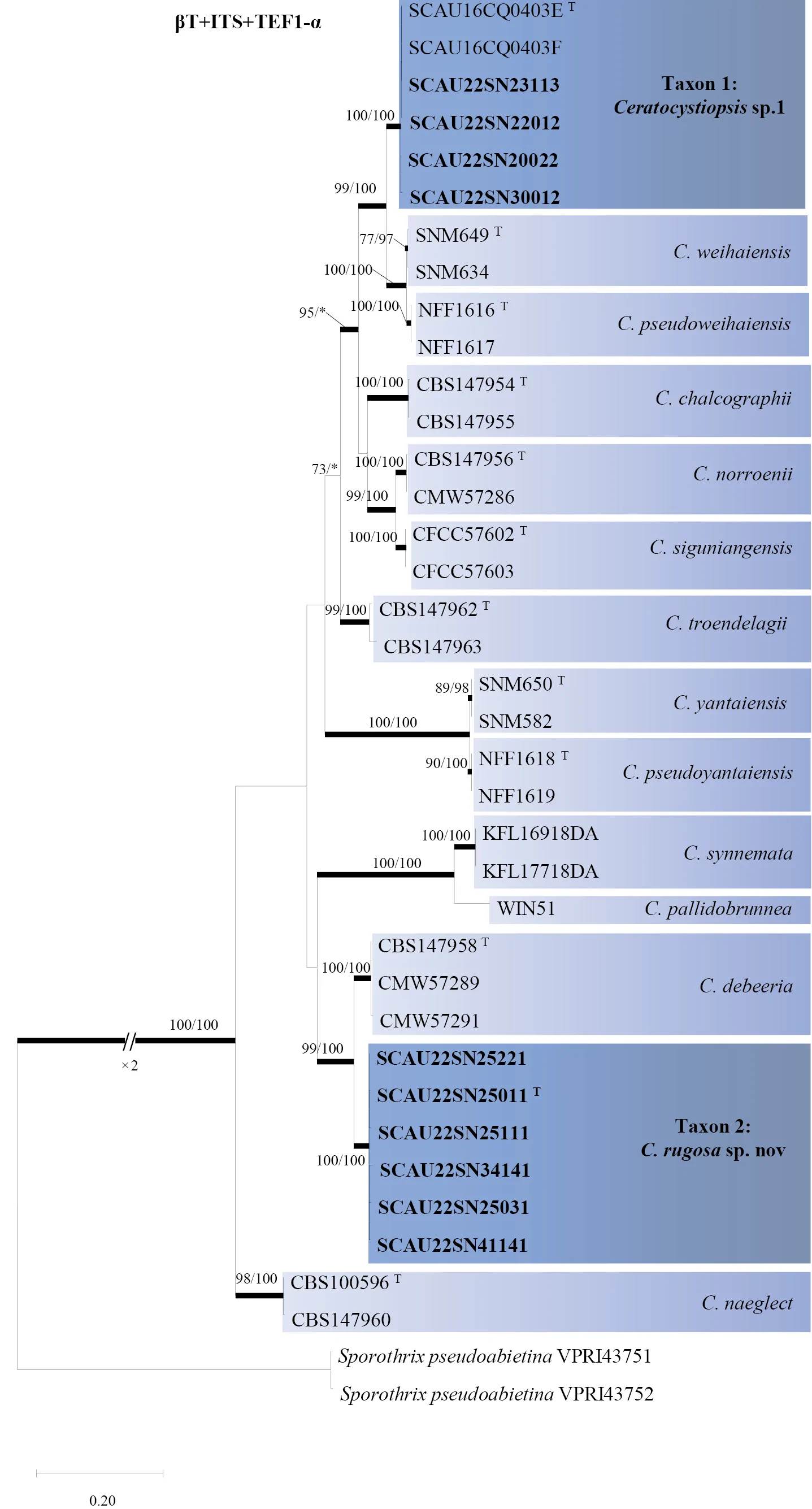
Maximum Likelihood tree of *Ceratocystiopsis* generated from the combined (βT + ITS + TEF1-α) sequence data. Sequences generated from this study are printed in bold. Bold branches indicate posterior probability values ≥ 0.95. Bootstrap values of ML/MP ≥ 70% are recorded at the nodes. T = ex-type isolates.

Phylogenetic inferences were constructed using βT (468 positions), ITS (634 positions), TEF1-α (625 positions), and the concatenated datasets (βT + ITS + TEF1-α, 1,727 positions). The best models of the four datasets for ML, MP, and BI analysis were estimated as GTR + G (βT dataset), TrN + G (ITS and TEF1-α datasets), and GTR + I + G (concatenated dataset). Based on the βT + ITS + TEF1-α phylogenetic tree (Fig. 3), our ten representative isolates formed two distinct, well-supported terminal clades, each representing a separate taxonomic unit. Four representative isolates of Taxon 1 formed a strongly supported lineage with unpublished strains obtained from GenBank (SCAU16CQ0403E and SCAU16CQ0403F); to avoid taxonomic controversy, these are not described as a new species here and are instead referred to as *Ceratocystiopsis* sp. 1. The six representative isolates of Taxon 2 formed a new clade. They were closely related to strains of the known species *C. debeeria* Jankow. & H. Solheim. Phylogenetic analyses based on βT, ITS, and TEF1-α consistently and robustly supported the distinction of Taxon 2 from related species (Suppl. material 1: figs S1, S2).

### Graphilbum

**Figure 4.**
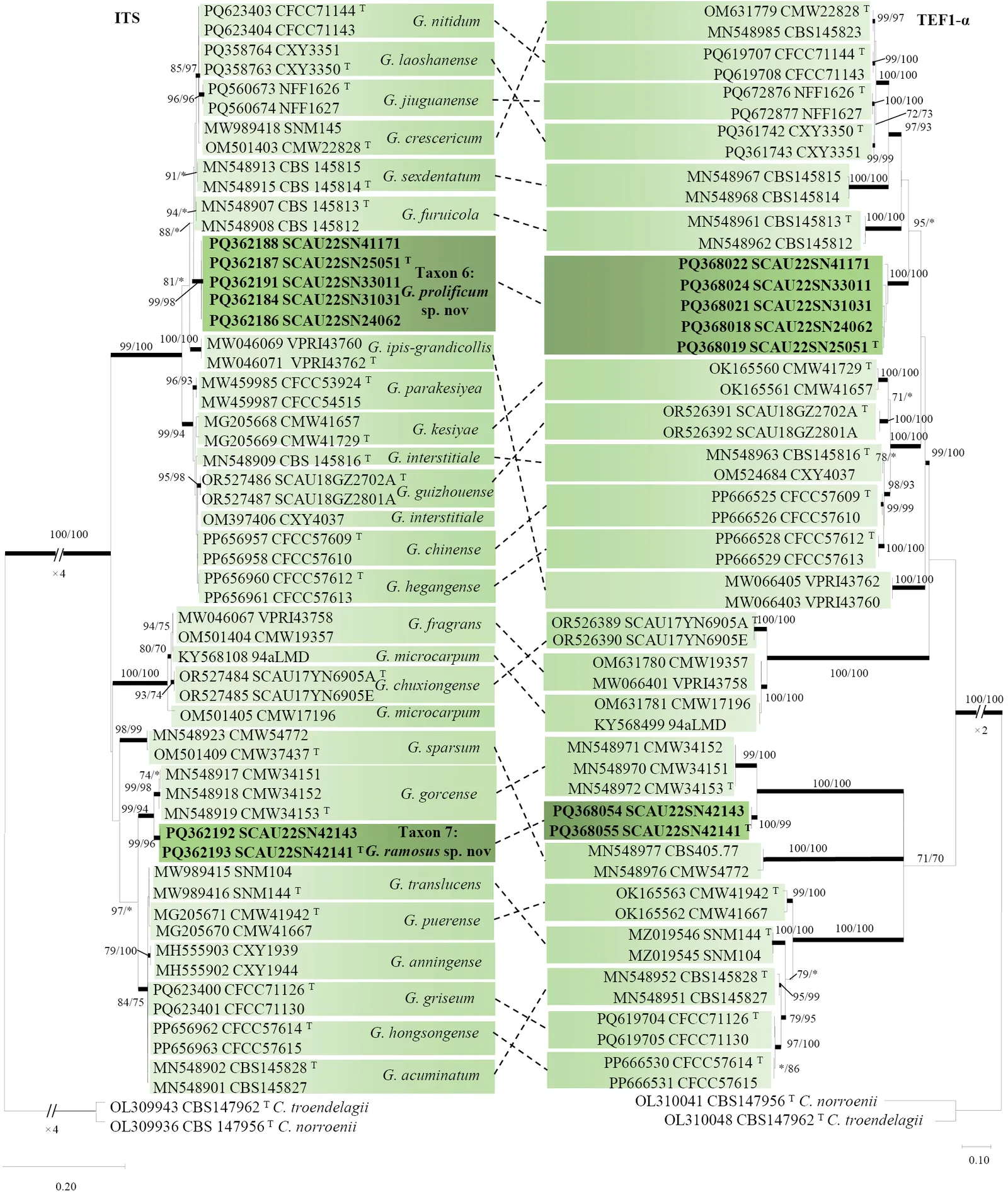
Maximum Likelihood tree of *Graphilbum* generated from the ITS and TEF1-α sequence data. Sequences generated from this study are printed in bold. Bold branches indicate posterior probability values ≥ 0.95. Bootstrap values of ML/MP ≥ 70% are recorded at the nodes. T = ex-type isolates.

In the genus *Graphilbum*, phylogenetic inferences were constructed using the βT (640 positions), ITS (592 positions), and TEF1-α (918 positions) datasets. The best models for the three datasets under ML, MP, and BI analyses were selected as GTR + I + G for the βT and ITS datasets, and TrN + I + G for the TEF1-α dataset. The three phylogenetic trees consistently resolved the interspecific relationships within the genus (Fig. 4; Suppl. material 1: fig. S3). Based on the ITS and TEF1-α phylogenetic trees, seven representative isolates were resolved into two distinct, well-supported terminal clades, each representing an undescribed taxon (Fig. 4). The five isolates of Taxon 6 were most closely related to the known species *G. crescericum* P. Romón, Z.W. de Beer, M.J. Wingf., *G. furuicola* Jankow. & H. Solheim, *G. jiuguanense* D. Xie, H.W. Chen & D.F. Chi, *G. laoshanense* Zheng Wang & Q. Lu, *G. nitidum* Zheng Wang & Q. Lu, and *G. sexdentatum* Jankow. & H. Solheim. The two isolates of Taxon 7 were closely related to the known species *G. gorcense* Marinc. & Jankow.

### *Leptographium* and Group A

**Figure 5.**
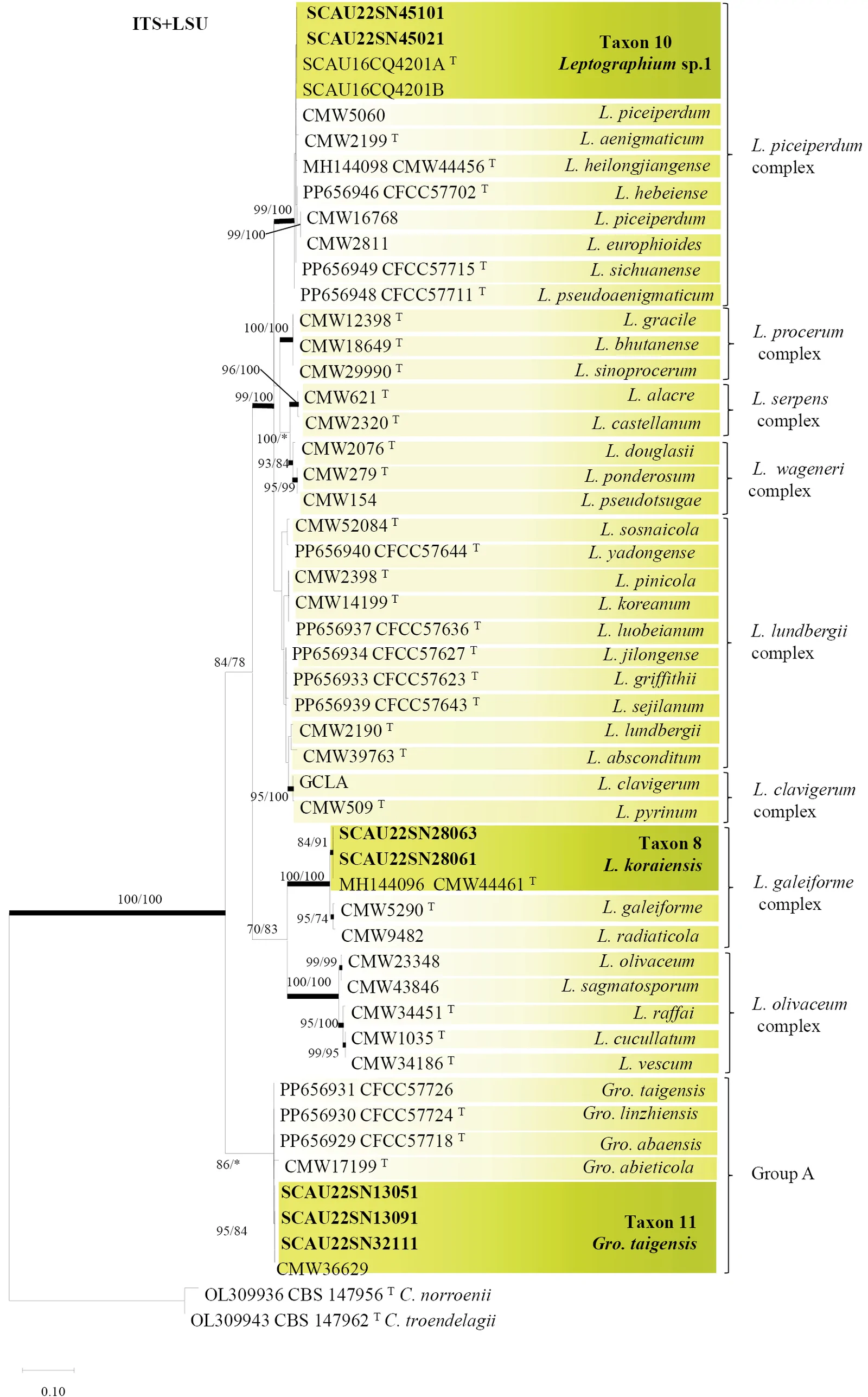
Maximum Likelihood tree of *Leptographium* and Group A generated from the ITS + LSU sequence data. Sequences generated from this study are printed in bold. Bold branches indicate posterior probability values ≥ 0.95. Bootstrap values of ML/MP ≥ 70% are recorded at the nodes. T = ex-type isolates.

The representative isolates of *Leptographium* and those of Group A were combined for phylogenetic analyses. This combined dataset was first subjected to phylogenetic inferences based on the βT (Fig. 2) (470 positions) and the combined ITS + LSU dataset (Fig. 5) (1,247 positions). The best models for the two datasets under ML, MP, and BI analyses were selected as TrN + I + G (βT dataset) and GTR + I + G (ITS + LSU dataset), respectively. The results indicated that the representative isolates of *Leptographium* belonged to three species complexes: *L. galeiforme*, *L. piceiperdum*, and *L. lundbergii*. For each of the three complexes and Group A, phylogenetic inferences were further conducted using the combined βT + TEF1-α dataset. The best models for the four combined βT + TEF1-α datasets under ML, MP, and BI analyses were selected as TrN + G (for the *L. galeiforme* complex dataset), GTR + I (for the *L. piceiperdum* complex dataset), and GTR + G (for both the *L. lundbergii* complex and Group A datasets).

**Figure 6.**
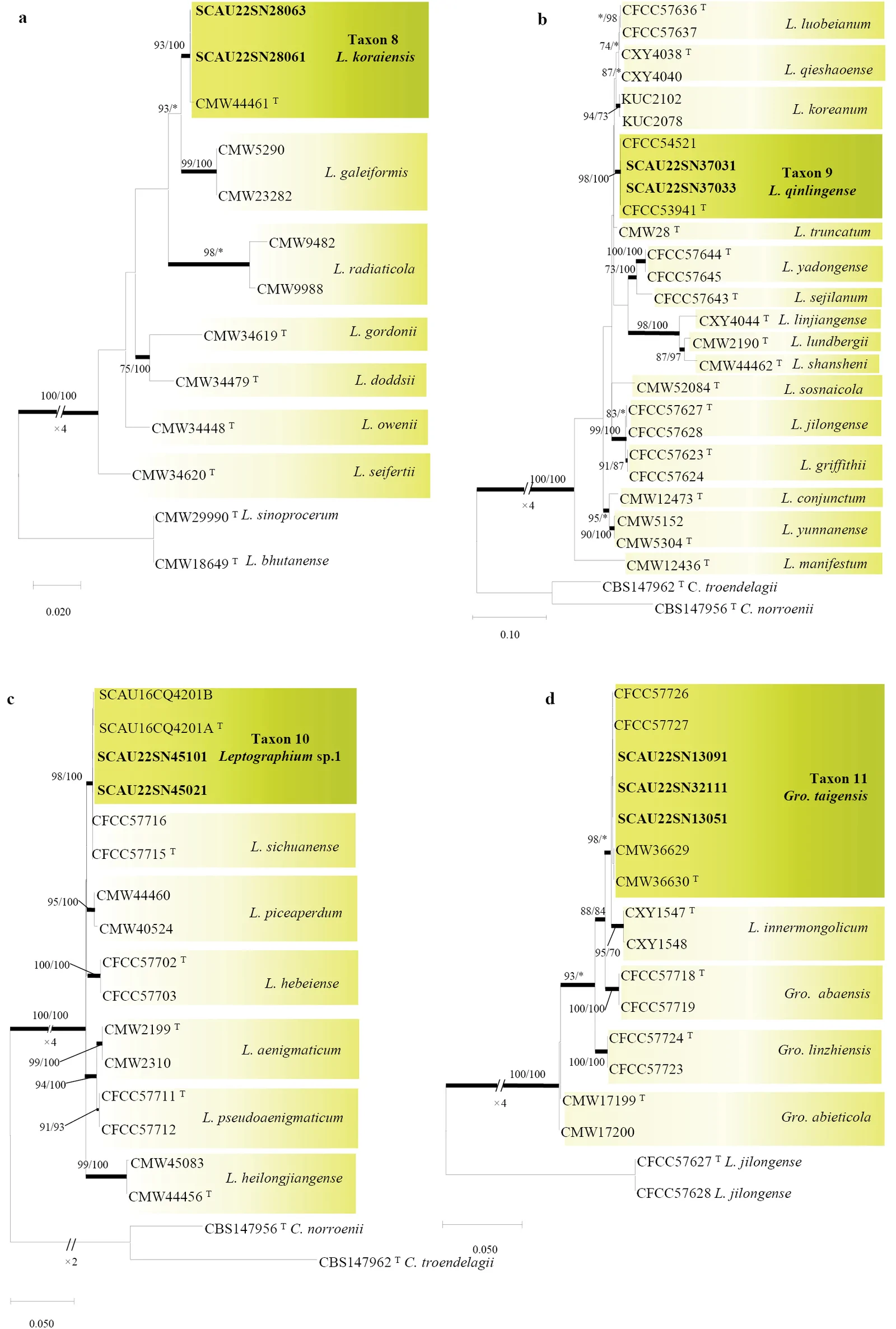
Maximum Likelihood tree of *Leptographium* and Group A generated from the combined (βT + TEF1-α) sequence data. **a**. *L. galeiforme* complex; **b**. *L. lundbergii* complex; **c**. *L. piceiperdum* complex; **d**. Group A. Sequences generated from this study are printed in bold. Bold branches indicate posterior probability values ≥ 0.95. Bootstrap values of ML/MP ≥ 70% are recorded at the nodes. T = ex-type isolates.

Phylogenetic analysis based on the combined dataset of the *L. galeiforme* complex (Fig. 6a; βT + TEF1-α, 637 positions) revealed that the two representative isolates of Taxon 8 clustered with the known species *L. koraiensis* R. Chang, Z.W. de Beer & M.J. Wingf., thus being identified as *L. koraiensis*. Phylogenetic analysis based on the combined dataset of the *L. lundbergii* complex (Fig. 6b; βT + TEF1-α, 1069 positions) indicated that the two representative isolates of Taxon 9 clustered with the known species *L. qinlingense* and were therefore identified as *L. qinlingense*. Phylogenetic analysis based on the combined dataset of the *L. piceiperdum* complex (Fig. 6c; βT + TEF1-α, 1,085 positions) showed that the two representative isolates of Taxon 10 formed a highly supported lineage with unpublished strains obtained from GenBank (SCAU16CQ4201A and SCAU16CQ4201B) and the known species *L. sichuanense* Zheng Wang & Q. Lu; to avoid taxonomic controversy, this lineage is provisionally referred to as *Leptographium* sp. 1. Phylogenetic analysis based on the combined dataset of Group A (Fig. 6d; βT + TEF1-α, 1,007 positions) revealed that the three representative isolates of Taxon 11 clustered with the known species *Grosmannia taigensis* (Linnak., Z.W. de Beer & M.J. Wingf.) M. Procter and Z.W. de Beer and were thus identified as *Gro. taigensis*.

### *Masuyamyces* and Group B

**Figure 7.**
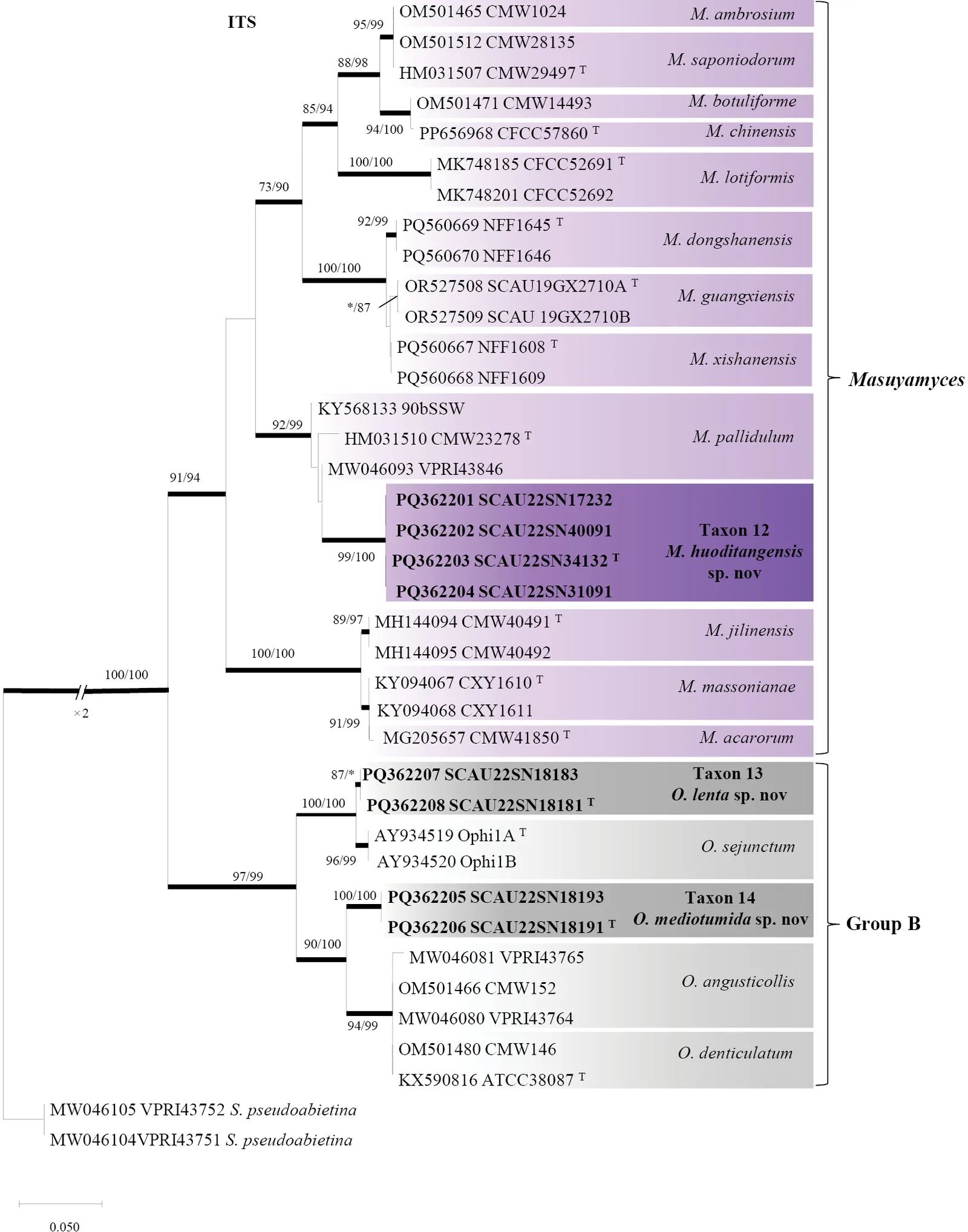
Maximum Likelihood tree of *Masuyamyces* and Group B generated from the ITS sequence data. Sequences generated from this study are printed in bold. Bold branches indicate posterior probability values ≥ 0.95. Bootstrap values of ML/MP ≥ 70% are recorded at the nodes (MP: Length = 416, CI = 0.752, RI = 0.938, RC = 0.706, HI = 0.248). T = ex-type isolates.

Within *Masuyamyces* and Group B, phylogenetic inferences were constructed using the βT (576 positions), ITS (539 positions), and TEF1-α (694 positions) datasets. The best models for the three datasets under ML, MP, and BI analyses were selected as TrN + I + G (for the βT dataset), GTR + G (for the ITS dataset), and GTR + I + G (for the TEF1-α dataset). Phylogenetic analyses revealed that our eight representative isolates were resolved into three well-supported clades (Fig. 7; Suppl. material 1: figs S4, S5), representing three undescribed taxa. Among these, Taxon 12 belongs to the genus *Masuyamyces*, while Taxa 13 and 14 belong to Group B. The four representative isolates of Taxon 12 were most closely related to *M. pallidulum* (Linnak., Z.W. de Beer & M.J. Wingf.) M. Procter and Z.W. de Beer; the two isolates of Taxon 13 formed a sister relationship with *O. sejunctum* M. Villarreal, Arenal, V. Rubio & De Troya; and the two isolates of Taxon 14 were most closely related to *O. angusticollis* (E.F. Wright & H.D. Griffin) M. Villarreal and *O. denticulatum* (R.W. Davidson) Z.W. de Beer & M.J. Wingf.

### *Ophiostoma clavatum* complex

**Figure 8.**
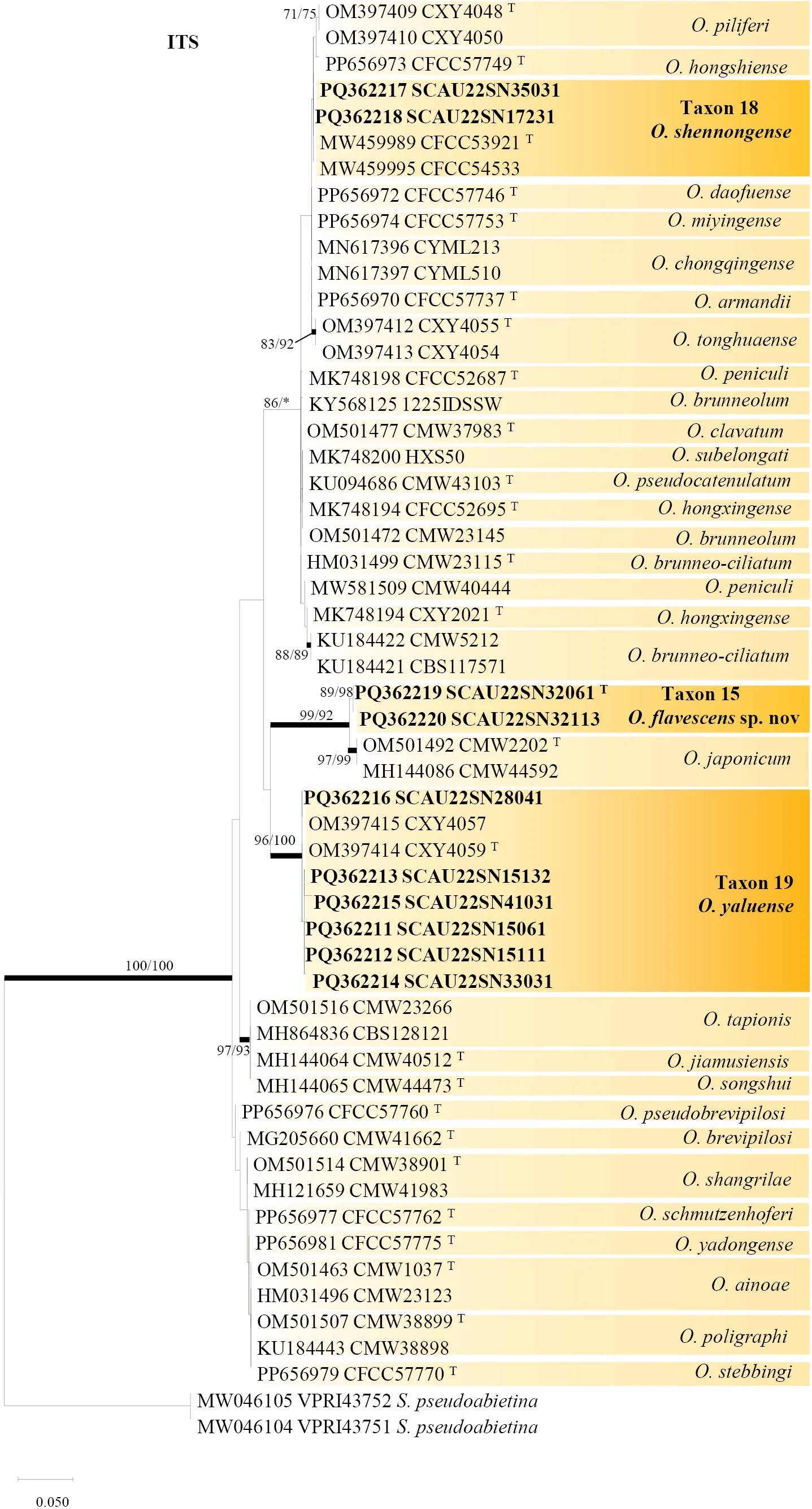
Maximum Likelihood tree of *O. clavatum* complex generated from the ITS sequence data. Sequences generated from this study are printed in bold. Bold branches indicate posterior probability values ≥ 0.95. Bootstrap values of ML/MP ≥ 70% are recorded at the nodes (MP: Length = 415, CI = 0.745, RI = 0.944, RC = 0.703, HI = 0.255). T = ex-type isolates.

**Figure 9.**
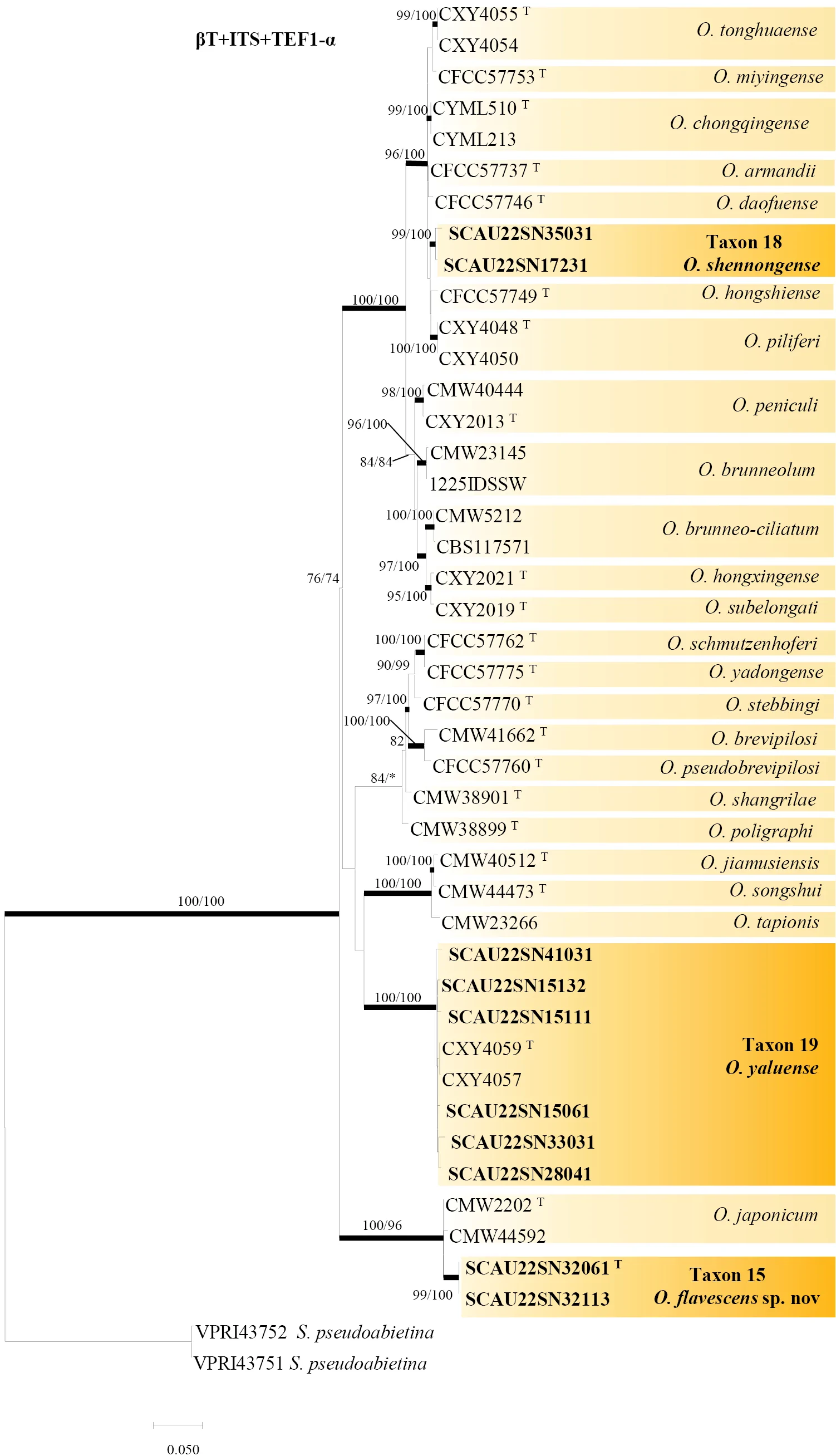
Maximum Likelihood tree of *O. clavatum* complex generated from the combined (βT + ITS + TEF1-α) sequence data. Sequences generated from this study are printed in bold. Bold branches indicate posterior probability values ≥ 0.95. Bootstrap values of ML/MP ≥ 70% are recorded at the nodes (MP: Length = 2050, CI = 0.663, RI = 0.890, RC = 0.590, HI = 0.337). T = ex-type isolates.

For this complex, phylogenetic inferences were constructed using the βT (345 positions), ITS (544 positions), TEF1-α (641 positions), and concatenated (βT + ITS + TEF1-α, 1,530 positions) datasets. The best models for the four datasets, as selected for ML, MP, and BI analyses, were GTR + I + G for the βT and concatenated datasets, TrN + G for the ITS dataset, and GTR + G for the TEF1-α dataset. Phylogenetic analyses showed that our ten representative isolates formed three distinct, well-supported terminal clades (Figs 8, 9; Suppl. material 1: fig. S6), representing *O. shennongense* (Taxon 18), *O. yaluense* H.M. Wang & Q. Lu (Taxon 19), and one undescribed taxon (Taxon 15). Taxon 15 was most closely related to the known species *O. japonicum* Yamaoka & M.J. Wingf.

## Taxonomy

Seven of the 20 taxa identified in this study were confirmed to represent different terminal clades and were interpreted as new species of *Ophiostomatales*.

### Ceratocystiopsis rugosa

Taxon classificationFungiOphiostomatalesOphiostomataceae

M.J.Chen & M.L.Yin sp. nov.

BA9B4A95-6F86-53BB-97D2-F9647DD87802

856450

Fig. 10

#### Etymology.

The epithet rugosa refers to the wrinkled surface of the colony.

**Figure 10.**
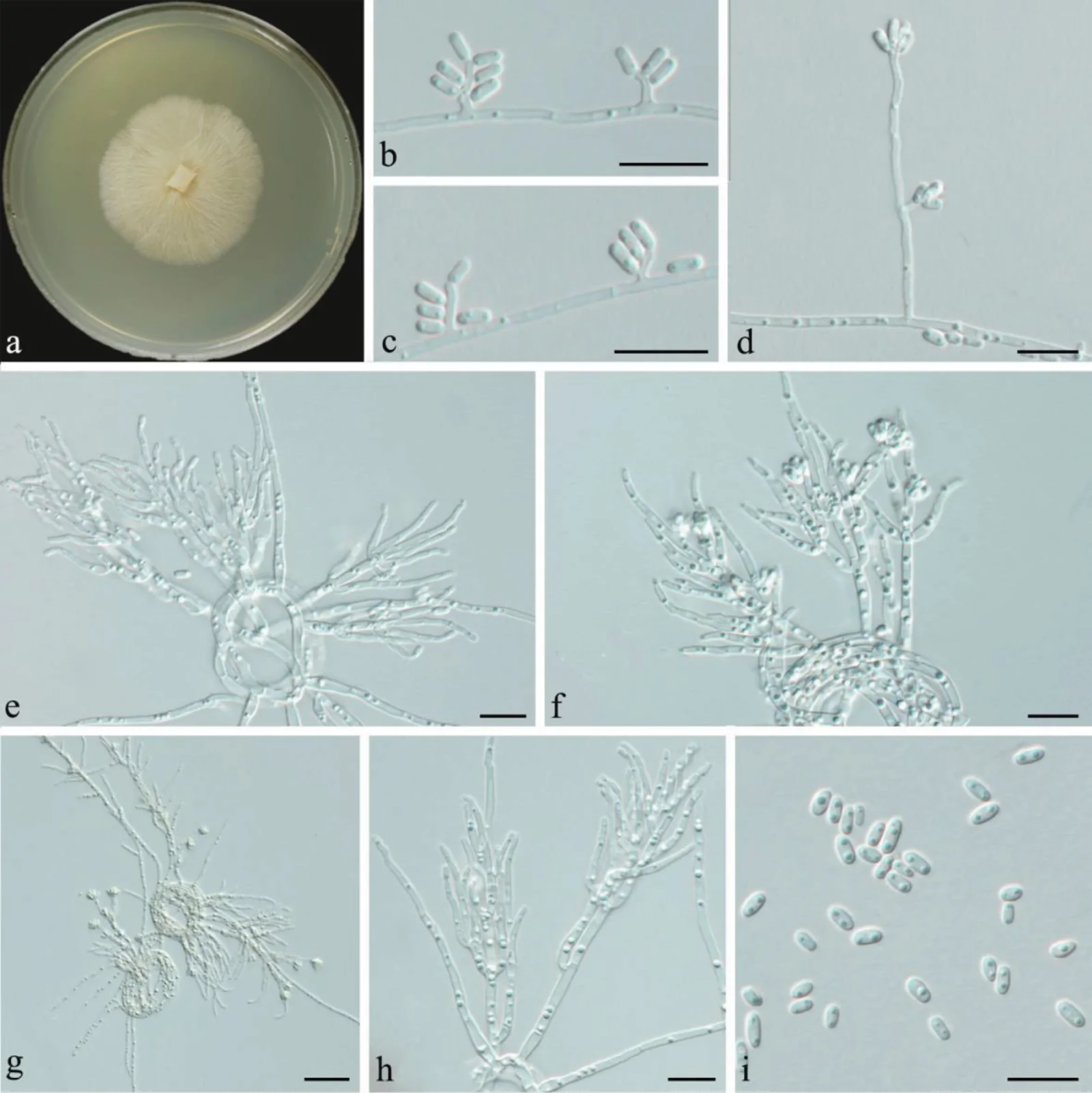
Morphological characteristics of *Ceratocystiopsis rugosa* sp. nov. (Taxon 2). **a**. Fourteen-day-old culture on 2% MEA; **b–d**. Simple and slightly branched micronematous conidiophores; **e–h**. Branching macronematous conidiophores; **i**. Conidia of *Hyalorhinocladiella*-like asexual morph. Scale bars: 5 μm (**b–i**).

#### Type.

China, • Shaanxi Province, Ankang City, collected from the gallery of *Polygraphus* sp. infesting *P. armandii*, Aug. 2022, Colls. M.J.Chen & M.L.Yin (holotype HAMS 352764; ex-holotype culture CGMCC 3.27261 = SCAU 22SN25011).

#### Description.

Sexual state not observed. ***Asexual state***: hyalorhinocladiella-like. Conidiophores micronematous or macronematous, abundantly formed. Micronematous conidiophores arising from vegetative hyphae are solitary, erect or slightly curved, transparent, usually producing 1–6 conidia measuring (1.5–) 2.0–4.6 (–6.4) × (0.4–) 0.5–0.7 (–0.8) μm. Macronematous conidiophores produced by coiled vegetative hyphae are erect, transparent, multibranched, (13.1–) 18.2–27.6 (–33.6) × (0.9–) –1.4 (–1.6) μm; conidia transparent, smooth, oblong or elliptic, (1.6–) 1.8–2.4 (–2.7) × (0.9–) –1.2 (–1.3) μm.

#### Culture characteristics.

Colonies on 2% MEA at 25 °C reaching a diameter of 41 mm in 14 days, yellowish white, surface rough and distinctly wrinkled, growing slowly, mycelium attached to the medium surface, and without aerial mycelium.

#### Insect vector.

*Polygraphus* sp.

#### Host trees.

*Pinus armandii*.

#### Distribution.

Ankang, Shaanxi Province.

#### Notes.

Phylogenetic analyses showed that *C. rugosa* is closely related to *C. debeeria*. The difference is that *C. rugosa* has smaller micronematous conidiophores and conidia than *C. debeeria*. In addition, the colony surface of *C. rugosa* is wrinkled, while that of *C. debeeria* is suede-like (Jankowiak et al. 2022).

#### Additional specimens examined.

China, • Shaanxi Province, Ankang, from galleries of *Polygraphus* sp. infesting *P. armandii*, Aug. 2022, Colls. M.J.Chen & M.L.Yin (culture SCAU 22SN25031 = SCAU 22SN25111 = SCAU 22SN25221 = SCAU 22SN34141 = SCAU 22SN41141).

### Graphilbum prolificum

Taxon classificationFungiOphiostomatalesOphiostomataceae

M.J.Chen & M.L.Yin sp. nov.

D80E2D6F-72B6-5FD5-929A-21620DBDDEF4

856451

Fig. 11

#### Etymology.

The epithet prolificum refers to the prolific nature of this fungus, which thrives and has strong sporulation ability.

**Figure 11.**
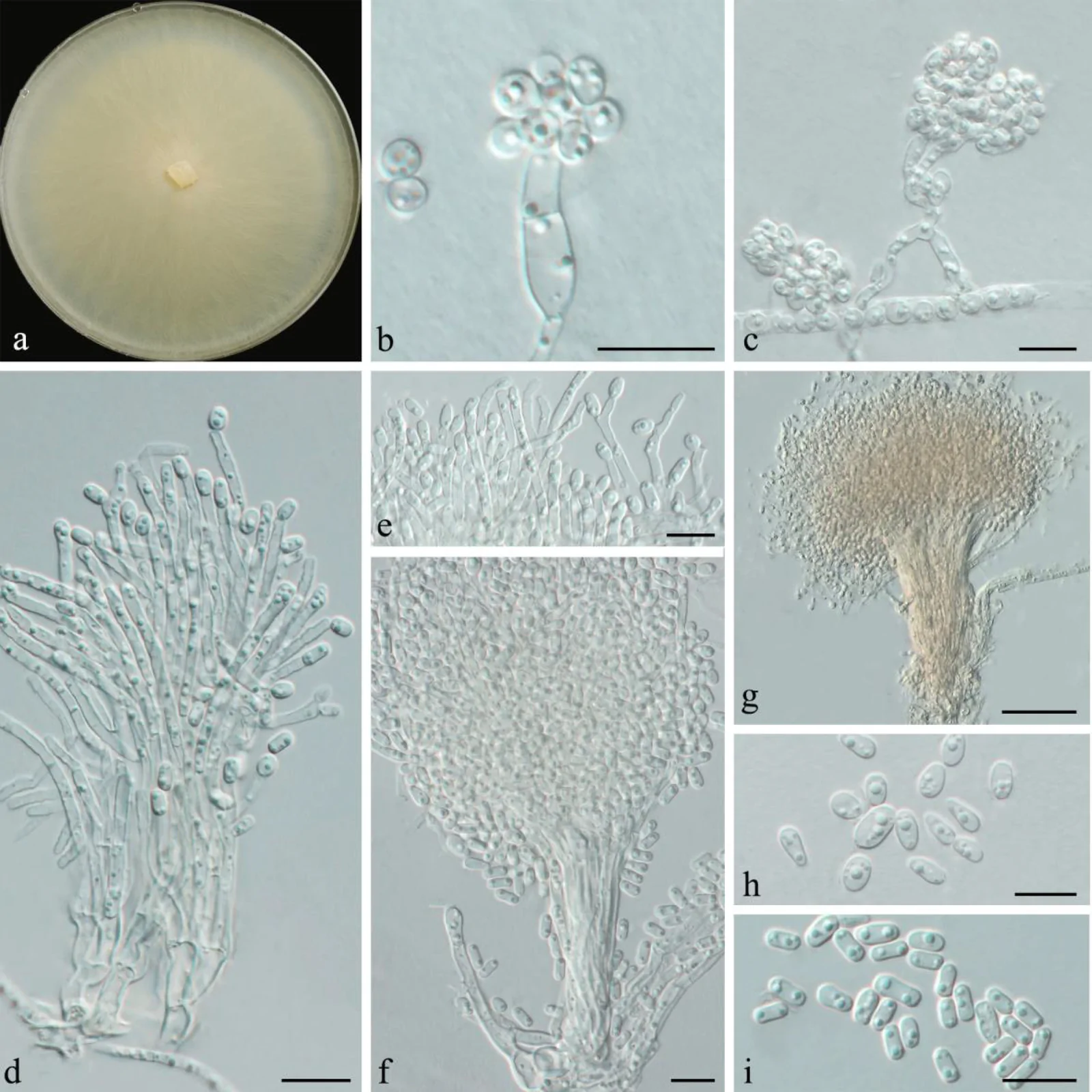
Morphological characteristics of *Graphilbum prolificum* sp. nov. (Taxon 6). **a**. Fourteen-day-old culture on 2% MEA; **b–d**. *Hyalorhinocladiella*-like asexual morph; **e**. Conidiogenous cells of *Pesotum*-like macronematal asexual morph; **f, g**. *Pesotum*-like macronematal asexual morph; **h**. Conidia of *Hyalorhinocladiella*-like asexual morph; **i**. Conidia of *Pesotum*-like asexual morph. Scale bars: 5 μm (**b, d–i**); 20 μm (**c**).

#### Type.

China, • Shaanxi Province, Ankang City, collected from the gallery of *Polygraphus* sp. infesting *P. armandii*, Aug. 2022, Colls. M.J.Chen & M.L.Yin (holotype HAMS 352765; ex-holotype culture CGMCC 3.27262 = SCAU 22SN25051).

#### Description.

Sexual state not observed. Asexual state: pesotum-like and hyalorhinocladiella-like.

#### Pesotum-like state.

Macronematous, usually single, sometimes in groups, erect, translucent, rod-shaped, loosely arranged, growing on the surface of pine wood or medium, (64.2–) 70.6–92.2 (–94.6) μm long, including meristem, base width (4.7–) 4.7–9.9 (–12.0) μm; conidiogenous cells are (9.2–) 10.8–17.8 (21.8–) × (0.9–) 0.9–1.1 (–1.2) μm, conidia are transparent, smooth, cylindrical to obovate, (2.0–) 2.2–2.8 (–3.4) × (0.9–) 0.9–1.3 (–1.9) μm.

#### Hyalorhinocladiella-like state.

Directly produced by mycelium submerged in the medium, conidiophores often 2–3 branches, transparent, erect, (26.7–) 28.8–49.2 (–47.5) × (1.5–) 1.5–1.9 (–2.0) μm; conidia are transparent, smooth, aseptate, oval to cylindrical, occasionally concentrated at the top of the conidiophores, forming raspberry-like, (1.6–) 1.8–2.2 (–2.4) × (1.0–) 1.1–1.3 (–1.6) μm.

#### Culture characteristics.

Colonies on 2% MEA at 25 °C reached a diameter of 79 mm in 7 days; white, circular, smooth margins; hyphae submerged in the medium; aerial hyphae sparse.

#### Insect vector.

*Polygraphus* sp.

#### Host trees.

*Pinus armandii*.

#### Distribution.

Ankang, Shaanxi Province.

#### Notes.

*Graphilbum prolificum* was phylogenetically closely related to *G. crescericum*, *G. furuicola*, *G. jiuguanense*, *G. laoshanense*, *G. nitidum* and *G. sexdentatum* (Fig. 4). These seven species also developed *pesotum*-like and/or *hyalorhinocladiella*-like asexual morphs. However, they differed in some morphological and cultural characteristics (Romón et al. 2014; Jankowiak et al. 2020; Chang et al. 2021; Song et al. 2024; Xie et al. 2025). Morphologically, the conidia of *G. prolificum* are considerably smaller than those of the other species. Culturally, *G. prolificum* (11.3 mm/d) grew more slowly than *G. jiuguanense* (12.3 mm/d), but faster than *G. crescericum* (7.6 mm/d), *G. furuicola* (2.5 mm/d), *G. laoshanense* (8.1 mm/d), *G. nitidum* (7.6 mm/d), and *G. sexdentatum* (5.1 mm/d). Based on both phylogenetic and morphological evidence, we propose recognizing *G. prolificum* as a novel species.

#### Additional specimens examined.

China, • Shaanxi Province, Ankang, from galleries of *Polygraphus* sp. infesting *P. armandii*, Aug. 2022, Colls. M.J.Chen & M.L.Yin (culture SCAU 22SN24062 = SCAU 22SN31031 = SCAU 22SN33011 = SCAU 22SN41171).

### Graphilbum ramosus

Taxon classificationFungiOphiostomatalesOphiostomataceae

M.J.Chen & M.L.Yin sp. nov.

F5D04983-B9EE-5782-B85D-D7016D0B5D50

856452

Fig. 12

#### Etymology.

The epithet ramosus refers to the conidiophores of this fungus, which often have many branches.

**Figure 12.**
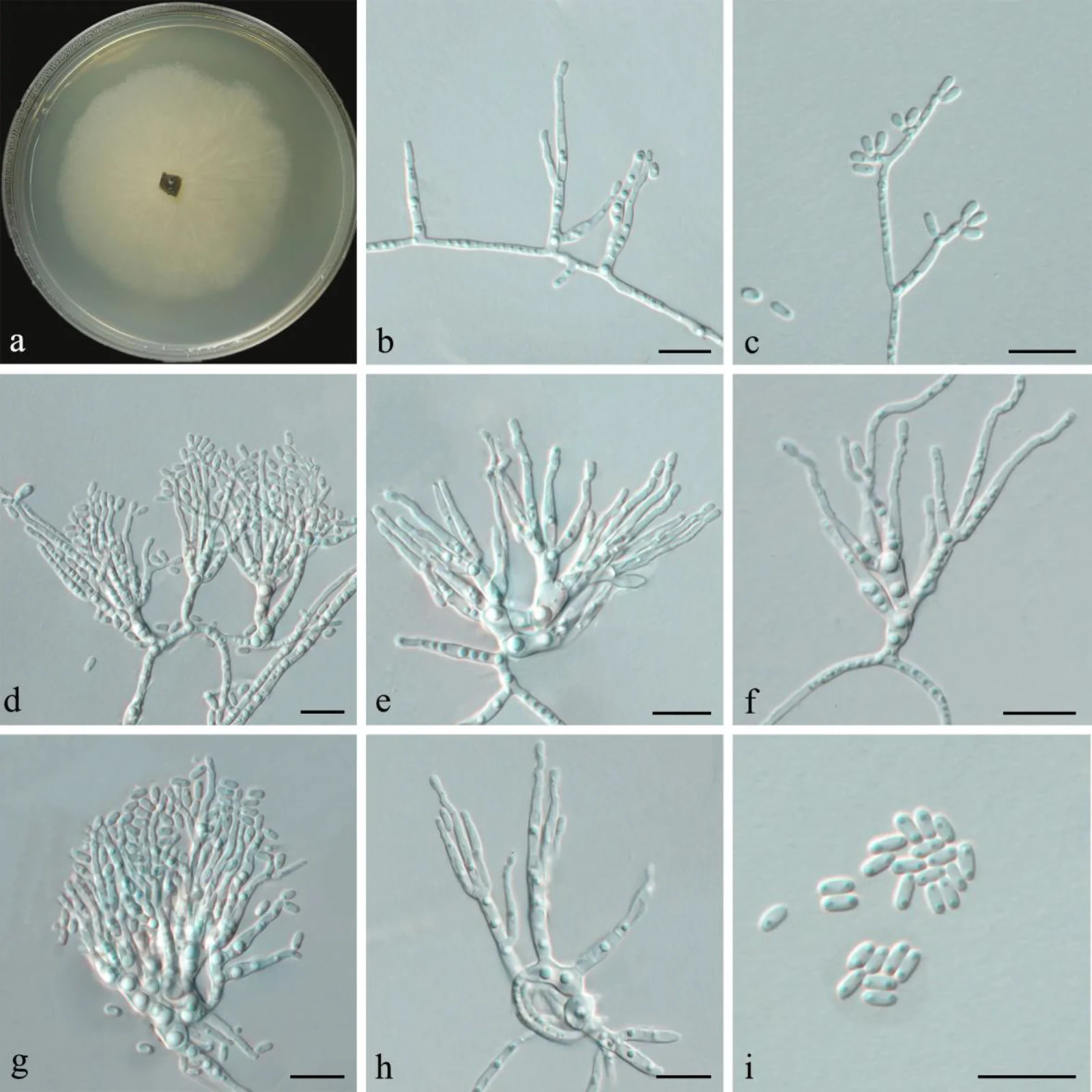
Morphological characteristics of *Graphilbum ramosus* sp. nov. (Taxon 7). **a**. Fourteen-day-old culture on 2% MEA; **b–h**. *Hyalorhinocladiella*-like asexual morph; **i**. Conidia of *Hyalorhinocladiella*-like asexual morph. Scale bars: 5 μm (**b–i**).

#### Type.

China, • Shaanxi Province, Ankang City, collected from the gallery of *D. armandi* infesting *P. armandii*, Aug. 2022, Colls. M.J.Chen & M.L.Yin (holotype HAMS 352766; ex-holotype culture CGMCC 3.27263 = SCAU 22SN42141).

#### Description.

Sexual state not observed. Asexual state: hyalorhinocladiella-like. Conidiophores directly produced by mycelium submerged in medium, transparent, usually strong branches, forming a dendritic shape, (13.6–) 15.9–22.3 (–25.8) μm long, (1.0–) 1.1–1.9 (–2.3) μm wide at the base. Conidia are oblong, transparent, smooth, (1.6–) 1.8–2.0 (–2.1) × (0.8–) 0.8–1.0 (–1.1) μm.

#### Culture characteristics.

Colonies on 2% MEA at 25 °C reached a diameter of 61 mm in 14 days; white, with hyphae submerged in agar and without aerial mycelium; later changing from white to pure black and staining the medium black after storage at 4 °C.

#### Insect vector.

*Dendroctonus armandi*.

#### Host trees.

*Pinus armandii*.

#### Distribution.

Ankang, Shaanxi Province.

#### Notes.

*Graphilbum gorcense* (Jankowiak et al. 2020) was closely related to *G. ramosus* in our phylogenetic analysis (Fig. 4). In terms of cultural characteristics, the colony of *G. gorcense* is white, becoming slightly greenish-gray with age, whereas *G. ramosus* does not exhibit this change. Furthermore, the growth rate of *G. gorcense* (5.9 mm/d) was faster than that of *G. ramosus* (4.4 mm/d) on 2% MEA at 25 °C. Morphologically, conidia of *G. gorcense* (3–)3.5–4(–4.5) × (1–)1.5–2(–2) μm are larger than those of *G. ramosus* (1.6–)1.8–2.0(–2.1) × (0.8–)0.8–1.0(–1.1) μm. Ecologically, *G. gorcense* was associated with *Tetropium* sp. infesting *Picea abies* (L.) H. Karst. in Poland, whereas *G. ramosus* was associated with *D. armandi* infesting *P. armandii* in Shaanxi Province, China.

#### Additional specimens examined.

China, • Shaanxi Province, Ankang, from galleries of *D. armandi* infesting *P. armandii*, Aug. 2022, Colls. M.J.Chen & M.L.Yin (culture SCAU 22SN42143).

### Masuyamyces huoditangensis

Taxon classificationFungiOphiostomatalesOphiostomataceae

M.J.Chen & M.L.Yin sp. nov.

43D577AC-B9B2-5AC9-8A41-2D357E0F6D99

856453

Fig. 13

#### Etymology.

The epithet huoditangensis refers to the forest farm of Huoditang, from which this fungus was collected.

**Figure 13.**
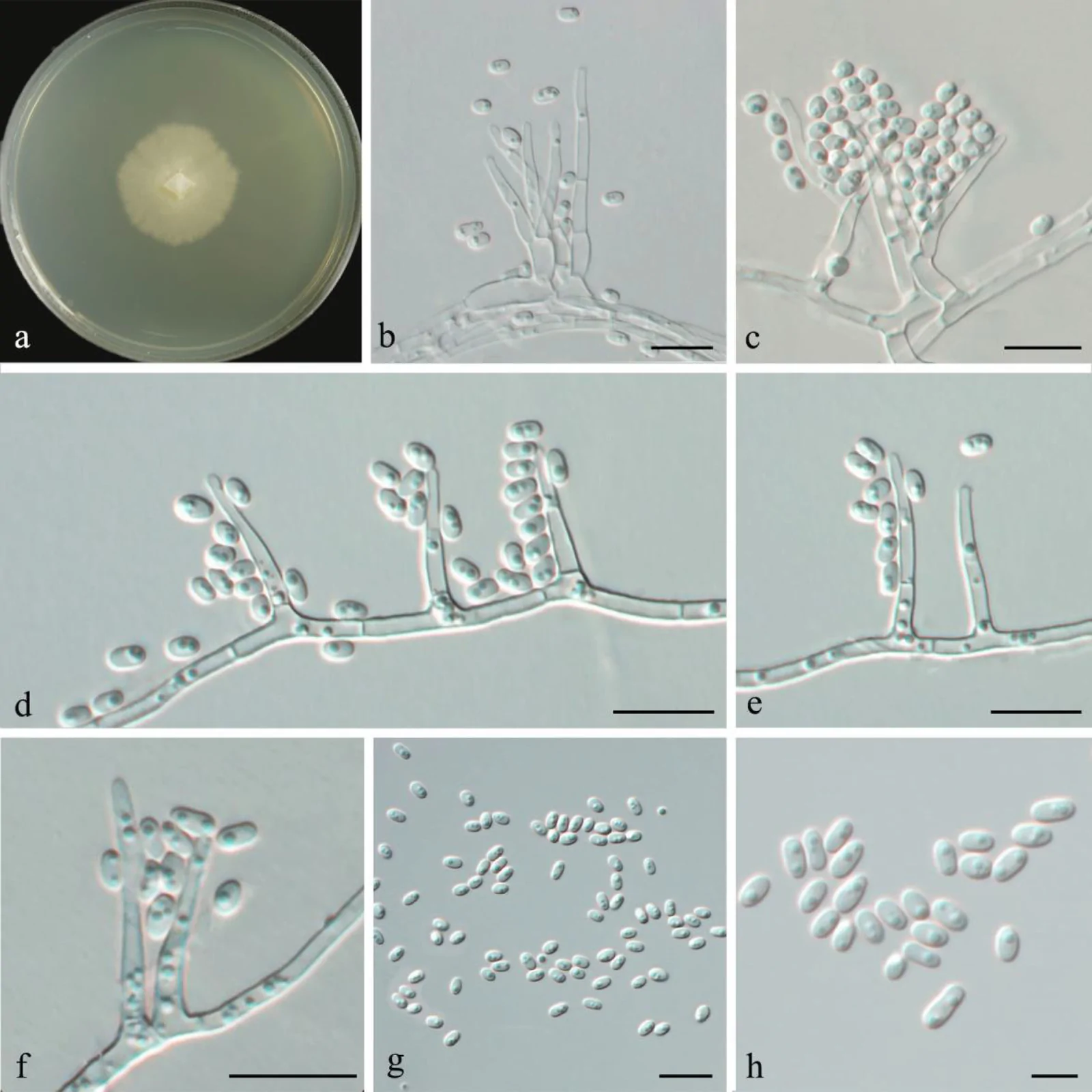
Morphological characteristics of *Masuyamyces huoditangensis* sp. nov. (Taxon 12). **a**. Fourteen-day-old culture on 2% MEA; **b–f**. *Hyalorhinocladiella*-like asexual morph; **g, h**. Conidia of *Hyalorhinocladiella*-like asexual morph. Scale bars: 5 μm (**b–h**).

#### Type.

China, • Shaanxi Province, Ankang City, collected from the gallery of *D. armandi* infesting *P. armandii*, Aug. 2022, Colls. M.J.Chen & M.L.Yin (holotype HAMS 352767; ex-holotype culture CGMCC 3.27264 = SCAU 22SN34132).

#### Description.

Sexual state not observed. Asexual state: hyalorhinocladiella-like. Conidiogenous cells arising directly from hyphae, transparent, septate, measuring (5.1–) 7.1–13.5 (–17.3) × (0.9–) –1.6 (–2) μm. Conidia are transparent, smooth, ovoid to oblong, (1.3–) 1.4–1.8 (–2.1) × (0.9–) 0.9–1.1 (–1.2) μm.

#### Culture characteristics.

Colonies on 2% MEA at 25 °C reaching a diameter of 30 mm in 14 days, white, surface smooth, growth slow, mycelium immersed in the medium, and without aerial mycelium.

#### Insect vector.

*Dendroctonus armandi*.

#### Host trees.

*Pinus armandii*.

#### Distribution.

Ankang, Shaanxi Province.

#### Notes.

In our phylogenetic analysis (Fig. 7), *M. huoditangensis* forms an independent clade within *Masuyamyces* and is closely related to *M. pallidulum*. Morphologically, although both species develop a hyalorhinocladiella-like asexual morph, the conidiophores and conidia of *M. huoditangensis* are smaller than those of *M. pallidulum*. In terms of cultural characteristics, colonies of *M. huoditangensis* are white, whereas those of *M. pallidulum* are hyaline; additionally, *M. huoditangensis* (2 mm/d) grows more slowly than *M. pallidulum* (6 mm/d) on 2% MEA at 25 °C. Ecologically, *M. huoditangensis* was associated with *D. armandi* on *P. armandii* in Shaanxi province, China. In contrast, *M. pallidulum* was associated with *Hylastes brunneus* Erichson, *Hylurgops palliatus* (Gyllenhal), *Tomicus minor* (Hartig), *Trypodendron lineatum* (Olivier), and *Dryocoetes autographus* (Ratzeburg) on *Pinus sylvestris* in Punkaharju, Finland (Linnakoski et al. 2010).

#### Additional specimens examined.

China, • Shaanxi Province, Ankang, from galleries of *D. armandi* infesting *P. armandii*, Aug. 2022, Colls. M.J.Chen & M.L.Yin (culture SCAU 22SN17232 = SCAU 22SN31091 = SCAU 22SN40091).

### Ophiostoma flavescens

Taxon classificationFungiRhabditidaCystidicolidae

M.J.Chen & M.L.Yin sp. nov.

F7F4F5E6-E4B6-581C-A008-A2BAC63E8416

856458

Fig. 14

#### Etymology.

The epithet flavescens, which means that the color of the middle of the colony is light yellow.

**Figure 14.**
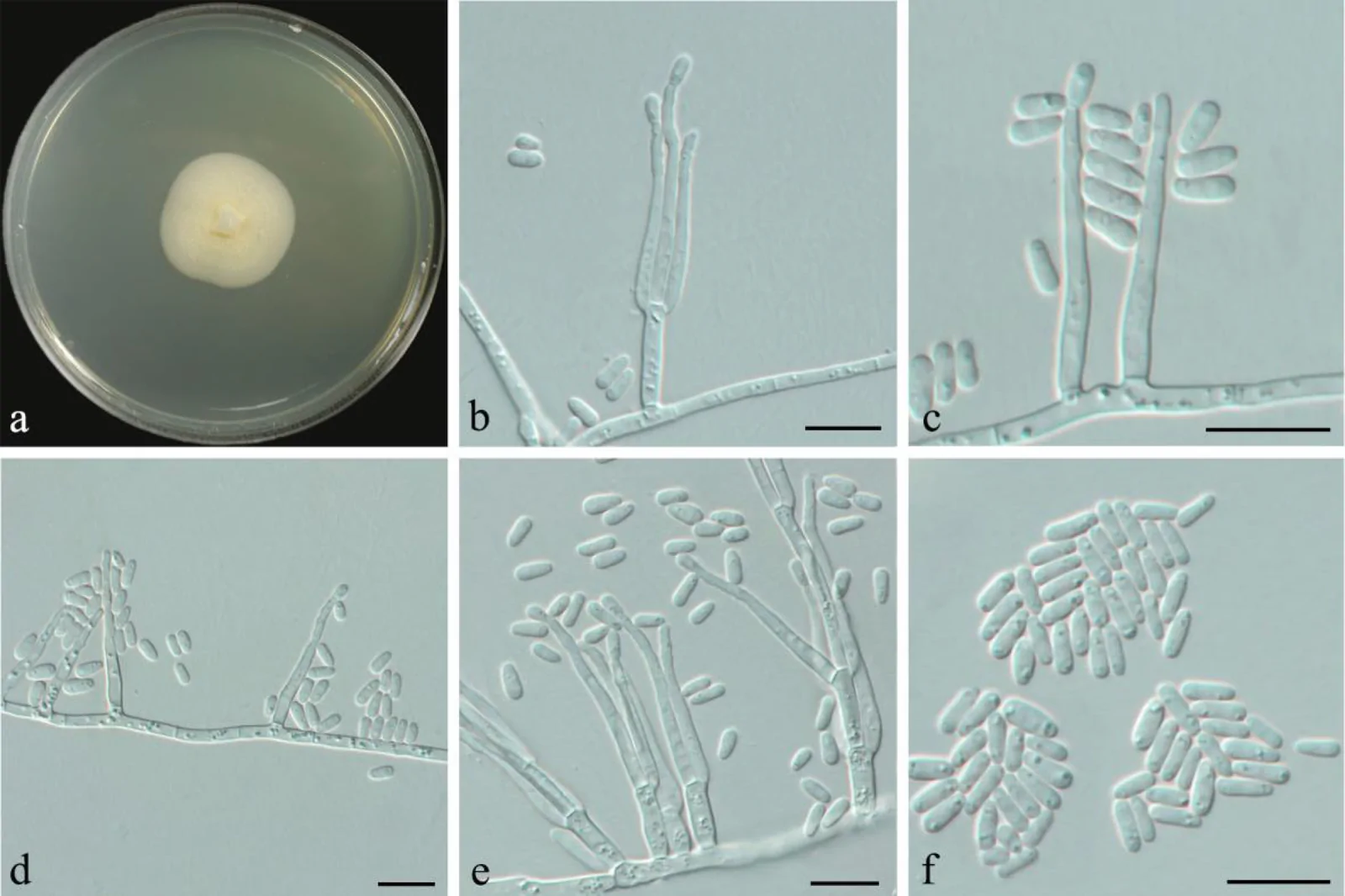
Morphological characteristics of *Ophiostoma flavescens* sp. nov. (Taxon 13). **a**. Fourteen-day-old culture on 2% MEA; **b–e**. *Hyalorhinocladiella*-like asexual morph; **f**. Conidia of *Hyalorhinocladiella*-like asexual morph. Scale bars: 5 μm (**b–f**).

#### Type.

China, • Shaanxi Province, Ankang City, collected from the gallery of *D. armandi* infesting *P. armandii*, Aug. 2022, Colls. M.J.Chen & M.L.Yin (holotype HAMS 352768; ex-holotype culture CGMCC 3.27265 = SCAU 22SN32061).

#### Description.

Sexual state not observed. Asexual state: hyalorhinocladiella-like. Conidiophores directly arise singly from the vegetative hyphae, or produce 1–3 branches, (10.3–) 14.0–25.0 (–37.5) × (0.9–) 1.1–1.5 (–1.7) μm. Conidia transparent, smooth, cylindrical or clavate, (1.9–) 2.4–3.0 (–3.2) × (0.9–) – 1.2 (–1.4) μm.

#### Culture characteristics.

Colonies on 2% MEA at 25 °C reach a diameter of 29 mm in 14 days; mycelium is immersed in the medium; margins are smooth; growth is slow; initially, light-yellow floccose mycelium is present at the center, and later the color gradually changes from light yellow to black.

#### Insect vector.

*Dendroctonus armandi*.

#### Host trees.

*Pinus armandii*.

#### Distribution.

Ankang, Shaanxi Province.

#### Notes.

*O. flavescens* was phylogenetically closely related to *O. japonicum* (Figs 8, 9). The difference is that *O. flavescens* lacks perithecia and has smaller conidia. In addition, the colony of *O. japonicum* is white at first, locally turning green with age (Yamaoka et al. 1997), while the colony of *O. flavescens* is light yellow at the beginning and locally turns black at a later stage.

#### Additional specimens examined.

China, • Shaanxi Province, Ankang, from galleries of *D. armandi* infesting *P. armandii*, Aug. 2022, Colls. M.J.Chen & M.L.Yin (culture SCAU 22SN32113).

### Ophiostoma lenta

Taxon classificationFungiRhabditidaCystidicolidae

M.J.Chen & M.L.Yin sp. nov.

EA89BBEE-CF2A-5422-ADA2-C332B7F9F2F7

856459

Fig. 15

#### Etymology.

The epithet lenta refers to the slow growth rate of the strain.

**Figure 15.**
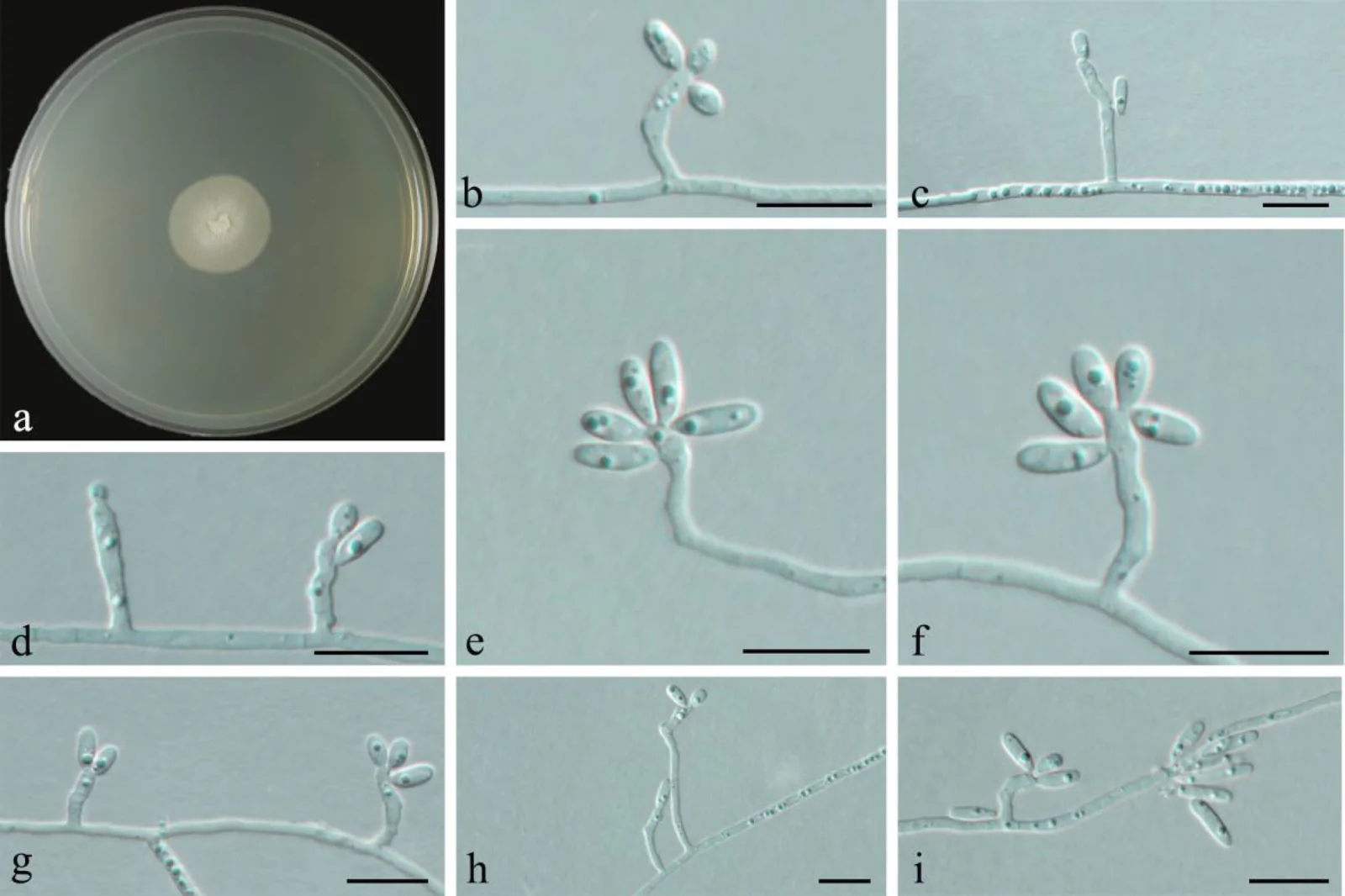
Morphological characteristics of *Ophiostoma lenta* sp. nov. (Taxon 15). **a**. Fourteen-day-old culture on 2% MEA; **b–i**. *Hyalorhinocladiella*-like asexual morph: conidiogenous cells and conidia. Scale bars: 5 μm (**b–i**).

#### Type.

China, • Shaanxi Province, Ankang City, collected from the gallery of *Dryocoetes hectographus* Reitter infesting *P. armandii*, Aug. 2022, Colls. M.J.Chen & M.L.Yin (holotype HAMS 352769; ex-holotype culture CGMCC 3.27266 = SCAU 22SN18181).

#### Description.

Sexual state not observed. Asexual state: hyalorhinocladiella-like. Conidiophores directly arising from the vegetative hyphae, conidiophores transparent, unbranched, straight or slightly curved, with rough edges, and the size is (2.5–) 3.8–9.2 (–14.3) × (0.6–) 0.7–0.9 (–1.1) μm. Conidia transparent, smooth, aseptate, clavate to ovate, (1.6–) 2.0–4.0 (–5.4) × (0.9–) –1.2 (–1.2) μm.

#### Culture characteristics.

Colonies on 2% MEA at 25 °C reach a diameter of 18 mm in 14 days; white, with color fading gradually from the center to the periphery; margins smooth; mycelium partially submerged in the medium; and without aerial mycelia.

#### Insect vector.

*Dryocoetes hectographus*.

#### Host trees.

*Pinus armandii*.

#### Distribution.

Ankang, Shaanxi Province.

#### Notes.

In our phylogenetic analysis, *O. sejunctum* and *O. lenta* formed sister clades (Fig. 7). The conidia of *O. sejunctum* are obovate to ellipsoidal, whereas the conidia of *O. lenta* are clavate to ovate and are generally smaller. In addition, *O. sejunctum* is characterized by the presence of allantoic ascospores and secondary and tertiary conidiophores, according to Villarreal et al. (2005). In contrast, *O. lenta* lacks these characteristics.

#### Additional specimens examined.

China, Shaanxi Province, Ankang, from galleries of *Dr. hectographus* infesting *P. armandii*, Aug. 2022, Colls. M.J.Chen & M.L.Yin (culture SCAU 22SN18183).

### Ophiostoma mediotumida

Taxon classificationFungiRhabditidaCystidicolidae

M.J.Chen & M.L.Yin sp. nov.

27DA7CE2-6ADC-54F0-A8D2-9FFC625F0E6C

856460

Fig. 16

#### Etymology.

The epithet mediotumida, medio means middle; tumida denotes a bulge, an enlarged part of the central part of the ascus.

**Figure 16.**
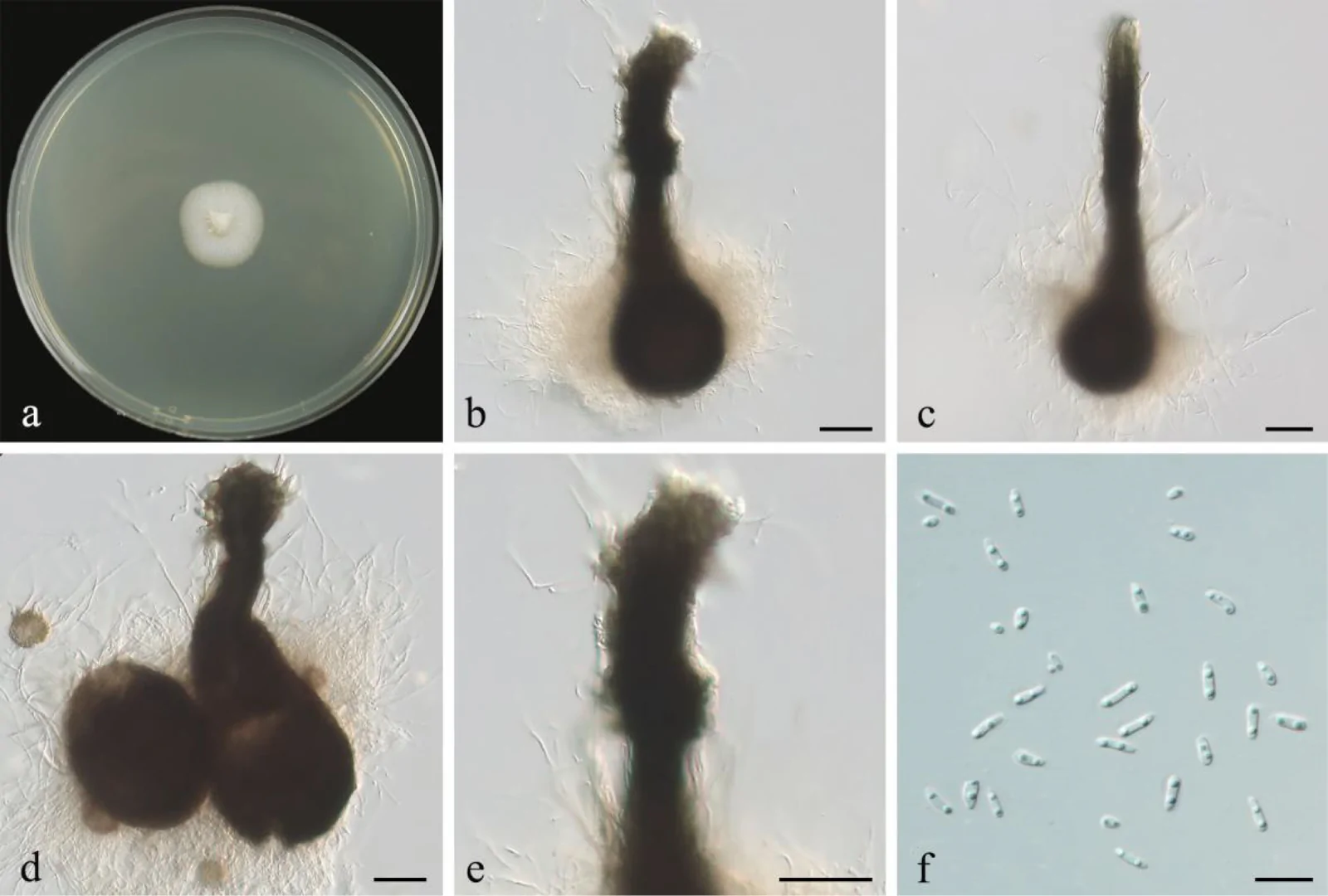
Morphological characteristics of *Ophiostoma mediotumida* sp. nov. (Taxon 16). **a**. Fourteen-day-old culture on 2% MEA; **b**–**d**. Perithecium; **e**. Apical part of the neck; **f**. Ascospores. Scale bars: 50 μm (**b–e**); 5 μm (**f**).

#### Type.

China, • Shaanxi Province, Ankang City, collected from the gallery of *Dr. hectographus* infesting *P. armandii*, Aug. 2022, Colls. M.J.Chen & M.L.Yin (holotype HAMS 352770; ex-holotype culture CGMCC 3.27267 = SCAU 22SN18191).

#### Description.

Asexual state not observed. Sexual state: perithecial. Perithecia appeared on the pine wood and grew on the surface of the agar or partially embedded in the agar. The base of the ascus shell was black, pear-shaped or water-drop-shaped, accompanied by transparent hyphae, with a diameter of (37.0–) 40.7–49.5 (–55.9) μm. Naturally extending out of the neck, straight or slightly curved, black, with a brush-like neck apex, accompanied by a ring-shaped bulge, full length (88.5–) 113.6–156.0 (–165.9) μm, no ostiolar hyphae. The basal width of the ascus neck is (13.8–) 15.4–20.4 (–23.7) μm, and the top width of the ascus neck is (10.0–) 11.0–13.8 (–14.7) μm. Ascospores hyaline, long striped, without sheath, aseptate, (1.7–) 2.4–3.4 (–4.0) × (0.6–) 0.9–1.1 (–1.2) μm.

#### Culture characteristics.

Colonies on 2% MEA at 25 °C reach a diameter of 19 mm in 14 days; growth is slow; the colony is milky white, with dense mycelium superficially adherent to the medium, and the margin is entire.

#### Insect vector.

*Dryocoetes hectographus*.

#### Host trees.

*Pinus armandii*.

#### Distribution.

Ankang, Shaanxi Province.

#### Notes.

Phylogenetic analysis revealed that *O. mediotumida* belongs to the currently undefined Group B and is closely related to *O. angusticollis* and *O. denticulatum* (Fig. 7). In terms of cultural characteristics, *O. angusticollis* appears hyaline to white and glossy on agar (Olchowecki and Reid 1974), whereas *O. mediotumida* is opaque milky white. Morphologically, *O. mediotumida* differs from *O. angusticollis* and *O. denticulatum* by lacking *sporothrix*-like asexual structures, and by having elongate ascospores instead of the kidney-shaped ascospores of *O. denticulatum* (De Beer et al. 2013b). Furthermore, in our study, *O. mediotumida* lacks a transparent gelatinous cushion at the apex of the ascus neck, and its ascospores are discharged in a filamentous manner from the neck apex.

#### Additional specimens examined.

China, • Shaanxi Province, Ankang, from galleries of *Dr. hectographus* infesting *P. armandii*, Aug. 2022, Colls. M.J.Chen & M.L.Yin (culture SCAU 22SN18193).

## Discussion

In the present study, 230 strains of ophiostomatalean fungi were isolated from the galleries of *P. armandii* infected by bark beetles. Combined with morphological and multi-gene phylogenetic analysis (βT, ITS, LSU, TEF1-α), 20 species belonging to six genera were identified. These included seven previously undescribed species. These new species are formally described as *C. rugosa*, *G. prolificum*, *G. ramosus*, *M. huoditangensis*, *O. flavescens*, *O. lenta* and *O. mediotumida*. In addition, because Taxon 1 and Taxon 10 showed high sequence similarity with unpublished strains from GenBank, we designated them as *Ceratocystiopsis* sp. 1 and *Leptographium* sp. 1, respectively, to avoid taxonomic controversy. In our study, the most abundant species were *O. shennongense*, followed by *O. ips*, *Ceratocystiopsis* sp. 1, *G. parakesiyea*, and *S. pseudoabietina*, which accounted for 27.39%, 11.30%, 9.13%, 9.13%, and 9.13% of all isolates, respectively (Table 2).

*O. shennongense* belongs to the *O. clavatum* complex and was previously discovered by Wang et al. (2022b), who confirmed it as the dominant species among the ophiostomatalean fungi associated with *D. armandi*. This finding is consistent with the results of the present study, indicating that the dominant association between *O. shennongense* and *D. armandi* is not accidental, and the reason may be attributable to the ability of this fungus to promote the colonization and development of *D. armandi*. The first ophiostomatalean fungi reported to be associated with *D. armandi* was *L. qinlingense* (Tang et al. 2004). In this study, only two strains of *L. qinlingense* were found. It had high pathogenicity and was a powerful assistant for *D. armandi* to invade *P. armandii* (Chen et al. 2004; Pham et al. 2014).

*Ceratocystiopsis* is widely distributed in Africa (Nel et al. 2021), Asia (Chang et al. 2021), and Europe (Jankowiak et al. 2022). To date, 38 species of the genus have been found. In this study, a total of three *Ceratocystiopsis* species were isolated. Among them, *C. weihaiensis* was first recorded in Shandong Province and previously reported to be associated with *Cryphalus piceae* (Ratzeburg) (Chang et al. 2021). However, in our study, *C. weihaiensis* was found to be associated with *Cyrtogenius luteus* (Blandford). This suggests that *C. weihaiensis* can associate with different bark beetle species across geographically distinct regions, providing clues for further investigation into its dispersal strategies and intraspecific genetic differentiation. *C. rugosa* has the closest genetic relationship with *C. debeeria*. *C. debeeria* was first reported by Jankowiak et al. (2022), and it was proposed that *C. debeeria* was a specific exosymbiont of *Pityogenes chalcographus* (Linnaeus). *Ceratocystiopsis* sp. 1 was isolated at a relatively high frequency in this study and was found to be associated with *D. armandi*, indicating that this unidentified species may occupy a relatively stable ecological niche in the gallery environment of *D. armandi* and may potentially play a facilitating role in the feeding and reproduction of the beetle. However, it remains unclear whether this fungus is pathogenic. Future studies should conduct artificial inoculation tests to evaluate the pathogenicity of *Ceratocystiopsis* sp. 1 to *P. armandii*.

*G. fragrans*, *G. parakesiyea*, *G. prolificum* and *G. ramosus* collected in this study belong to the genus *Graphilbum*, which has expanded to 36 formally described species with a wide distribution. This genus has been reported in Africa (Kamgan et al. 2008), Asia (Chang et al. 2017; Wang et al. 2019), Europe (Jankowiak et al. 2020), and the Americas (Davidson 1971; Geldenhuis et al. 2004; Reid and Hausner 2015). *Graphilbum* spp. are mainly characterized by synnematous *Pesotum*-like and mononematous *Hyalorhinocladiella*-like asexual morphs (De Beer and Wingfield 2013a; Jankowiak et al. 2020). *G. prolificum* is most closely related to the evolutionary branches composed of *G. crescericum*, *G. furuicola*, *G. jiuguanense*, *G. laoshanense*, *G. nitidum* and *G. sexdentatum*, and the morphological differences between them are very weak, which can be distinguished by the differences in molecular sequences (Romón et al. 2014; Jankowiak et al. 2020; Chang et al. 2021; Song et al. 2024; Xie et al. 2025). *G. ramosus* is closely related to *G. gorcense*, which was found to be related to *Tetropium* sp. by Jankowiak et al. (2020). In the genus *Graphilbum*, *G. gorcense* is the only species that does not form synnemata and is the only species that produces gray-green colonies. Interestingly, no synnemata were found in *G. ramosus* in this study, either. *G. fragrans* is the most common species within the genus and has been collected from various pine bark beetles and weevils across Canada (Jacobs et al. 2003), Europe (Mathiesen-Käärik 1953; Romón et al. 2007), the USA, New Zealand, Australia (Harrington et al. 2001), South Africa (Zhou et al. 2006), Japan (Masuya et al. 2009), and Korea (Kim et al. 2007). In China, this fungus has previously been recorded from *Tomicus yunnanensis* Kirkendall & Faccoli on *P. yunnanensis* Franch., *Pissodes* spp. on *Tsuga dumosa* (D. Don) Eichler and *P. armandii*, as well as from galleries of *T. minor* on *P. kesiya* Royle ex Gordon (Paciura et al. 2010; Zhou et al. 2013; Chang et al. 2017). This study confirms the association between *G. parakesiyea* and *P. armandii* for the second time; however, unlike previous reports, *G. parakesiyea* was isolated from *P. armandii* infested by *Polygraphus* sp. rather than from that infested by *D. armandi* (Wang et al. 2022b).

*L. koraiensis* belongs to the *L. galeiforme* complex and was first reported to be associated with *Ips typographus* in Heilongjiang Province (Chang et al. 2019). In this study, only two strains of *L. koraiensis* were isolated, and they were related to *D. armandi*. *Leptographium* sp.1 is located in the *L. piceiperdum* complex, in which all species have *Leptographium*-like asexual morphology and cup-shaped ascospores (De Beer and Wingfield 2013a). *Gro. taigensis* resides in an independent lineage within the *Ophiostomatales* that has not yet been assigned to any known species complex; in this study, this lineage is tentatively designated as Group A. This species was first discovered in Russia, where it was found to be associated with a variety of conifer-infesting bark beetles (Linnakoski et al. 2012). Liu et al. (2017) discovered this species in Inner Mongolia, which was the first record in China. Subsequently, Chang et al. (2017, 2019) isolated this fungus from *Ips subelongatus* Motschulsky and *Ips typographus* in Inner Mongolia and Jilin. Previous studies showed that this species was weakly toxic to *Larix* species, so we speculated that *Gro. taigensis* was also weakly toxic to *P. armandii* and could assist various bark beetles in the region in attacking *P. armandii* (Wang et al. 2021).

*M. huoditangensis* is closely related to *M. pallidulum*, and they can be well distinguished by phylogenetic analysis. *M. pallidulum* was discovered by Linnakoski et al. (2010) in pine and spruce trees in Finland, mainly related to *Hylastes brunneus*. Later, Jankowiak and Bilański (2013) demonstrated that *M. pallidulum* could live as a saprophyte in the roots of dead *Pinus sylvestris* independently of bark beetles infecting pine trees.

*O. ips* belongs to the *O. ips* complex (De Beer and Wingfield 2013a). Species in the *Ophiostoma* genus were characterized by cylindrical or allantoic ascospores with pillow-like sheaths and produced a series of asexual forms, which were described as *Hyalorhinocladiella*-like, *Leptographium*-like and *Pesotum*-like (Zipfel et al. 2006; Linnakoski et al. 2010). *O. ips*, one of the most frequently isolated ophiostomatalean fungi in China, was associated with various bark beetles that widely infect conifers (Lu et al. 2009; Chang et al. 2017; Wang et al. 2018), causing serious damage to conifer species in China. *O. flavescens* is closely related to *O. japonicum*. It was first discovered and reported in Japan as related to *Ips typographus* (Yamaoka et al. 1997). Later, Chang et al. (2019) also found and reported that *O. japonicum* was related to *Ips typographus* in Heilongjiang. These species could be distinguished not only by phylogenetic analysis but also by morphological data. For example, *O. flavescens* did not have asci *in vitro* culture and had smaller conidia. *O. yaluense* has been reported in association with *Tomicus pilifer* (Spessivtsev) infesting *P. koraiensis* Siebold & Zucc. in northeastern China (Wang et al. 2022a). In the present study, a total of 11 strains of *O. yaluense* were isolated, marking the first record of this fungus in Shaanxi Province, where it is associated with *D. armandi* infesting *P. armandii*.

In this study, *O. lenta* and *O. mediotumida* were placed in an independent lineage within the *Ophiostomatales* that has not yet been included in any recognized species complex. Here we designate this lineage as Group B (Chang et al. 2017; Wang et al. 2020; Trollip et al. 2021). *O. mediotumida* is closely related to *O. angusticollis* and *O. denticulatum*, and *O. angusticollis* has been reported to be associated with *Dendroctonus adjunctus* Blandford in Spain (Pérez Vera et al. 2011). Phylogenetic analysis of ITS showed that *O. lenta* is closely related to *O. sejunctum* (Fig. 7). *O. sejunctum* was known to be from Spain and related to *Tomicus piniperda* (Linnaeus) on *P. pinaster* Aiton (Villarreal et al. 2005). Since this species had only an ITS sequence and lacked sequencing of other gene fragments, we distinguished it based on morphological differences. *O. sejunctum* was characterized by allantoic ascospores and the presence of secondary and tertiary conidiophores, but no sexual structure was found in *O. lenta*; instead, only *Hyalorhinocladiella*-like conidiophores and a small number of long striped conidia were found.

In our research, we also recorded the known species *O. minus* in the *O. minus* complex (Gorton et al. 2004), which was widely distributed and recorded in the Northern Hemisphere and China. It was one of the most important fungi that cause blue wood stain (Gorton and Webber 2000; Lu et al. 2009). In previous studies, the *O. minus* lineage was divided into two allopatric populations, the Eurasian branch and the North American branch. Later, *O. minus* found in ophiostomatalean fungi associated with *Tomicus* species in southwestern China was divided into a third allopatric population (Gorton et al. 2004; Wang et al. 2019, 2020). Different studies have shown that the Eurasian branch often combined with *Tomicus piniperda* or various bark beetles to kill endangered pine trees (Gorton and Webber 2000; Wang et al. 2020), while *O. minus* of the North American branch often interacted with *Dendroctonus frontalis* Zimmerman (Barras and Perry 1972). In the present study, we found that *O. minus* and the third allopatric population aggregated on the same branches, so we speculated that this population could synergize with multiple bark beetles to damage pine trees. Of course, the origin, global distribution, and relationship of these populations to bark beetles still require further study.

In the present study, *S. pseudoabietina* was identified as a member of the *S. gossypina* complex, a group that is commonly associated with diverse bark beetles and mites (De Beer et al. 2016). This species was first discovered by Wang et al. (2019) and isolated from the adults of *T. yunnanensis* and *T. minor*. Later, the first record of *S. pseudoabietina* spread by *Xyleborus* was found in Australia. Studies have shown that this species has weak pathogenicity and does not show the potential to become the main pathogen of pine plants (Mahony et al. 2023).

## Conclusions

In this study, we collected samples from Chengguan Town and Huoditang Forest Farm in Shaanxi Province. A total of seven species from five genera were collected from Chengguan Town, and fifteen species from six genera were collected from Huoditang Forest Farm. Based on the phylogenetic data in this study, all ophiostomatalean fungi obtained from Chengguan Town are known species. In contrast, we found seven previously undescribed species in the ophiostomatalean fungi collected from Huoditang Forest Farm, indicating that the ophiostomatalean fungi resources in this area have great potential for further discovery. Although this study found many ophiostomatalean fungi in the galleries of bark beetles, the symbiotic mechanism between ophiostomatalean fungi and bark beetles, as well as the pathogenicity of the new species, still requires further study.

## Supplementary Material

XML Treatment for Ceratocystiopsis rugosa

XML Treatment for Graphilbum prolificum

XML Treatment for Graphilbum ramosus

XML Treatment for Masuyamyces huoditangensis

XML Treatment for Ophiostoma flavescens

XML Treatment for Ophiostoma lenta

XML Treatment for Ophiostoma mediotumida

## Additional information

### Artificial Intelligence (AI) use

The authors accept full responsibility for the content of the manuscript, including the disclosure of any use of AI.

No AI tools were used in the preparation of this manuscript.

### Author ORCIDs

M. Chen https://orcid.org/0009-0008-5611-5057

Y. Ran https://orcid.org/0009-0006-5726-8667

C. Liu https://orcid.org/0009-0009-6335-3149

M. Yin https://orcid.org/0000-0002-8811-3151

### Data availability

The manuscript mentions all available data. Ex-type cultures of new species were deposited in the Culture Collection of South China Agricultural University (SCAU) and the China General Microbiological Culture Collection Center (CGMCC). The type herbaria were preserved in the Fungarium (HAMS), Institute of Microbiology, Chinese Academy of Sciences. DNA sequence data are available in GenBank (https://www.ncbi.nlm.nih.gov/nucleotide/), and taxonomic novelties are available in MycoBank (https://www.mycobank.org/).

## Supplementary materials

Supplementary material 1 Supplementary figures Data type pdf Explanation note **fig. S1**. Maximum Likelihood tree of *Ceratocystiopsis* generated from the βT sequence data. Sequences generated from this study are printed in bold. Bold branches indicate posterior probability values ≥ 0.95. Bootstrap values of ML/MP ≥ 70% are recorded at the nodes. T = ex-type isolates. **fig. S2**. Maximum Likelihood tree of *Ceratocystiopsis* generated from the ITS and TEF1-α sequence data. Sequences generated from this study are printed in bold. Bold branches indicate posterior probability values ≥ 0.95. Bootstrap values of ML/MP ≥ 70% are recorded at the nodes. T = ex-type isolates. **fig. S3**. Maximum Likelihood tree of *Graphilbum* generated from the βT sequence data. Sequences generated from this study are printed in bold. Bold branches indicate posterior probability values ≥ 0.95. Bootstrap values of ML/MP ≥ 70% are recorded at the nodes. T = ex-type isolates. **fig. S4**. Maximum Likelihood tree of *Masuyamyces* and Group B generated from the βT sequence data. Sequences generated from this study are printed in bold. Bold branches indicate posterior probability values ≥ 0.95. Bootstrap values of ML/MP ≥ 70% are recorded at the nodes. T = ex-type isolates. **fig. S5**. Maximum Likelihood tree of *Masuyamyces* and Group B generated from the TEF1-α sequence data. Sequences generated from this study are printed in bold. Bold branches indicate posterior probability values ≥ 0.95. Bootstrap values of ML/MP ≥ 70% are recorded at the nodes. T = ex-type isolates. **fig. S6**. Maximum Likelihood tree of *O. clavatum* complex generated from the βT and TEF1-α sequence data. Sequences generated from this study are printed in bold. Bold branches indicate posterior probability values ≥ 0.95. Bootstrap values of ML/MP ≥ 70% are recorded at the nodes. T = ex-type isolates. https://binary.pensoft.net/file/1779681This dataset is made available under the Open Database License (http://opendatacommons.org/licenses/odbl/1.0/). The Open Database License (ODbL) is a license agreement intended to allow users to freely share, modify, and use this Dataset while maintaining this same freedom for others, provided that the original source and author(s) are credited. Minjie Chen, Yutong Ran, Congwang Liu, Mingliang Yin
