# Supplementary material for: Ophiostomatalean fungi associated with bark beetles infesting Pinus armandii Franch., with characterizations of seven new species from Shaanxi, China
Source: MycoKeys. 2026 Sep 18;140:173–216. doi: 10.3897/mycokeys.140.199146 (PMC13615396; doi:10.3897/mycokeys.140.199146)
Supplement: Supplementary file 1 [file mycokeys-140-173-s001.pdf]

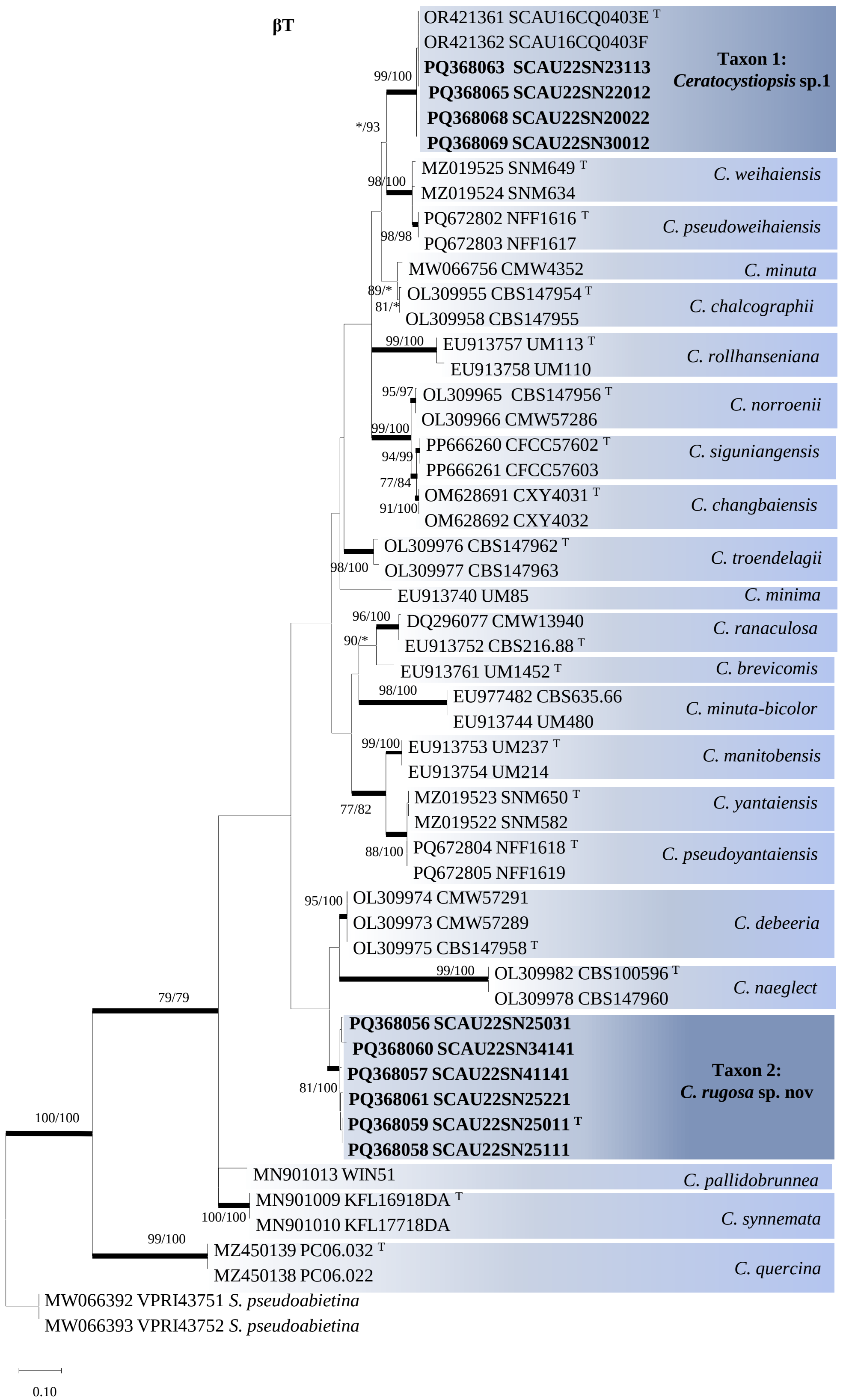

S1. Maximum Likelihood tree of *Ceratocystiopsis* generated from the βT sequence data. Sequences generated from this study are printed in bold. Bold branches indicate posterior probability values ≥ 0.95. Bootstrap values of ML/MP ≥ 70% are recorded at the nodes. T = ex-type isolates.

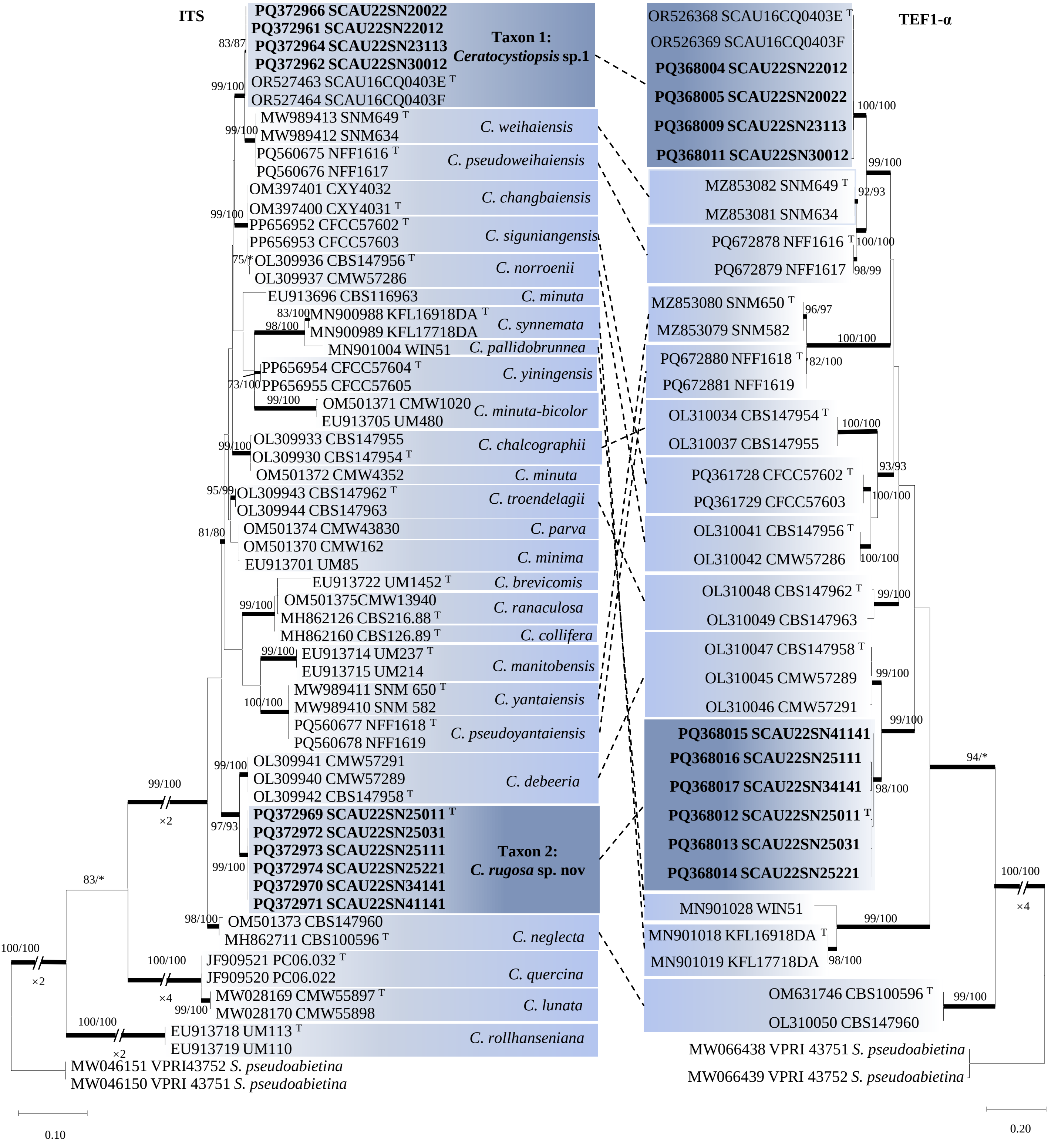

S2. Maximum Likelihood tree of *Ceratocystiopsis* generated from the ITS and TEF1-α sequence data. Sequences generated from this study are printed in bold. Bold branches indicate posterior probability values ≥ 0.95. Bootstrap values of ML/MP ≥ 70% are recorded at the nodes. T = ex-type isolates.

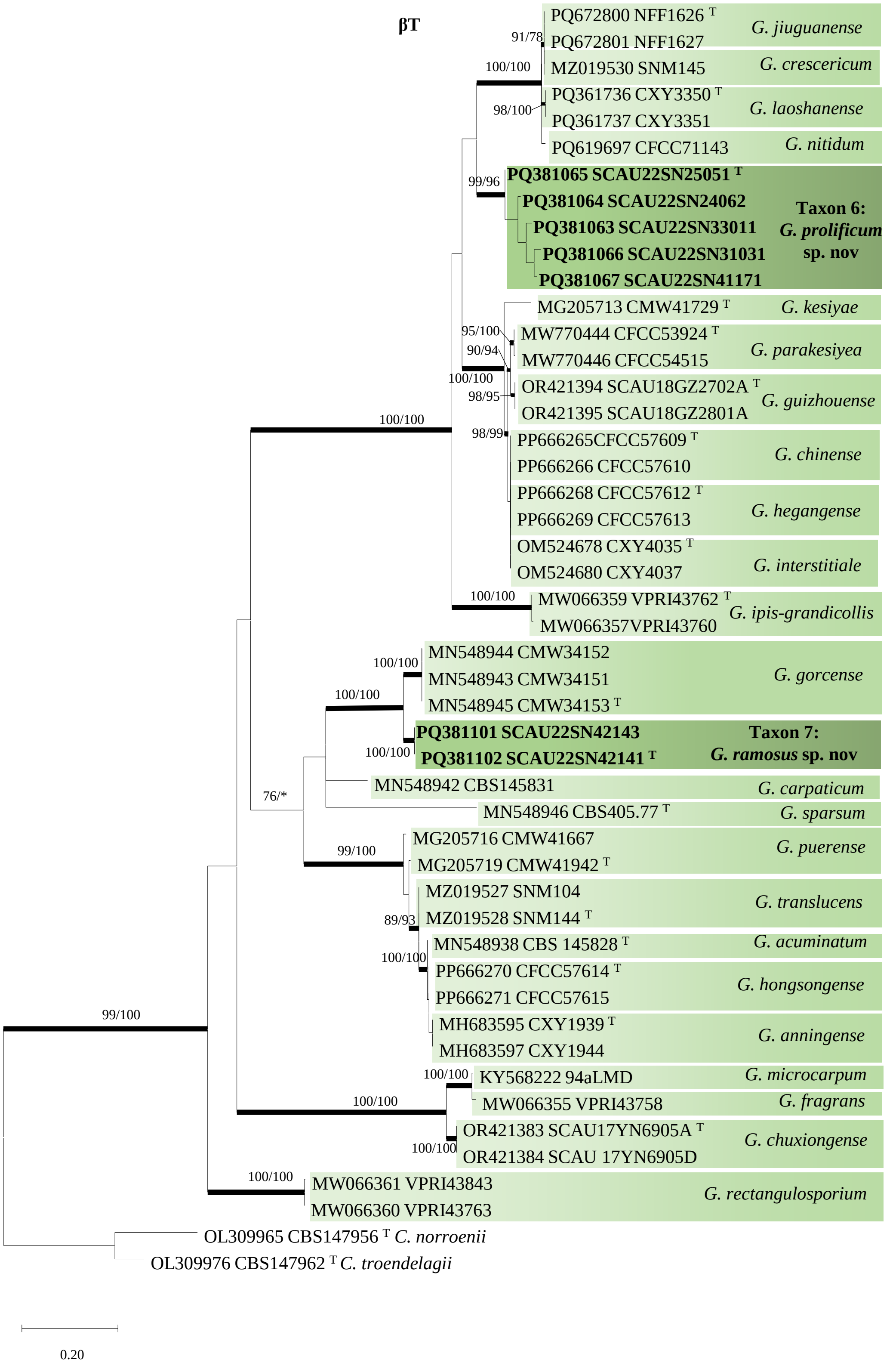

S3. Maximum Likelihood tree of *Graphilbum* generated from the βT sequence data. Sequences generated from this study are printed in bold. Bold branches indicate posterior probability values ≥ 0.95. Bootstrap values of ML/MP ≥ 70% are recorded at the nodes. T = ex-type isolates.

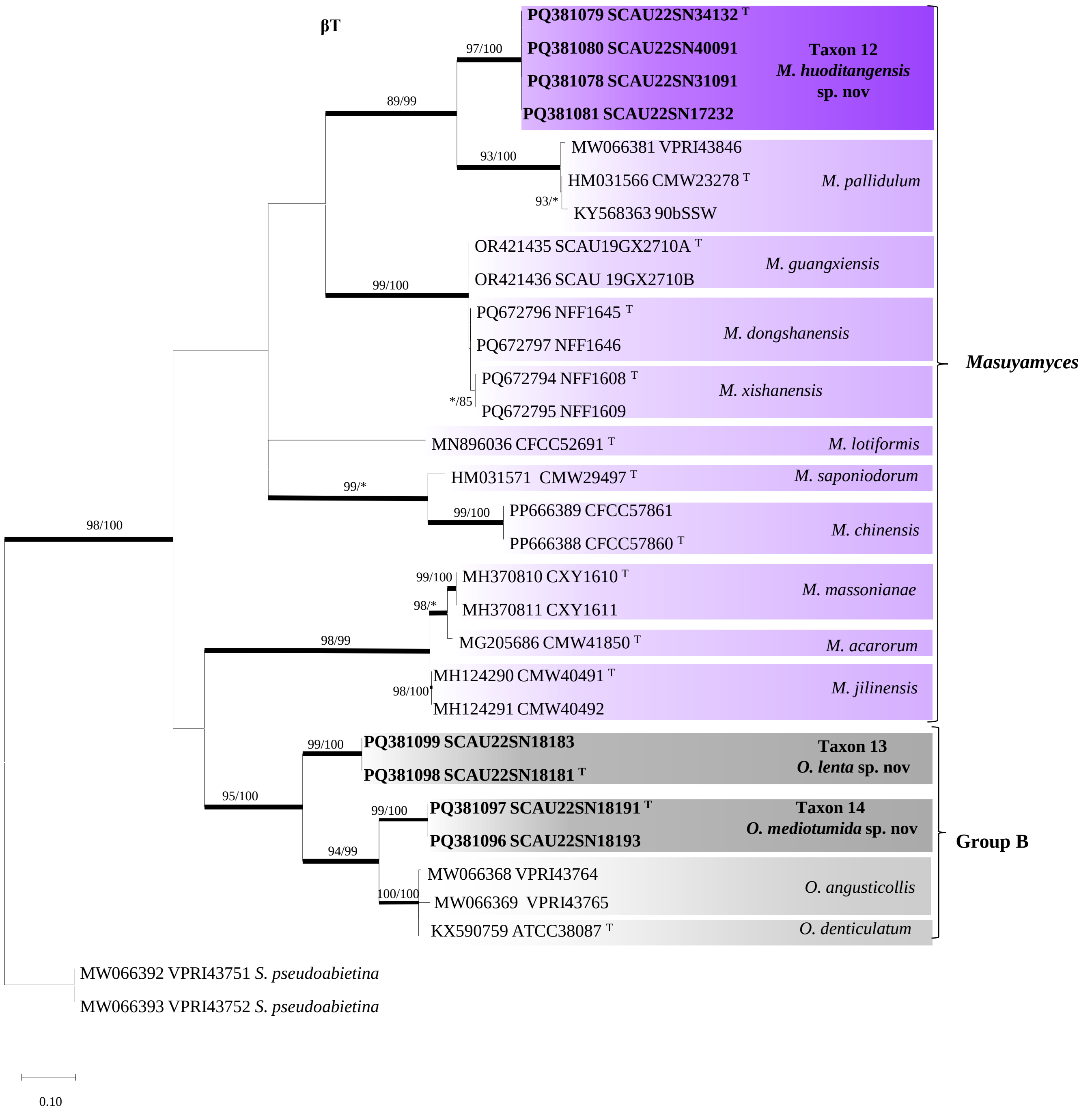

S4. Maximum Likelihood tree of *Masuyamyces* and Group B generated from the βT sequence data. Sequences generated from this study are printed in bold. Bold branches indicate posterior probability values ≥ 0.95. Bootstrap values of ML/MP ≥ 70% are recorded at the nodes. T = ex-type isolates.

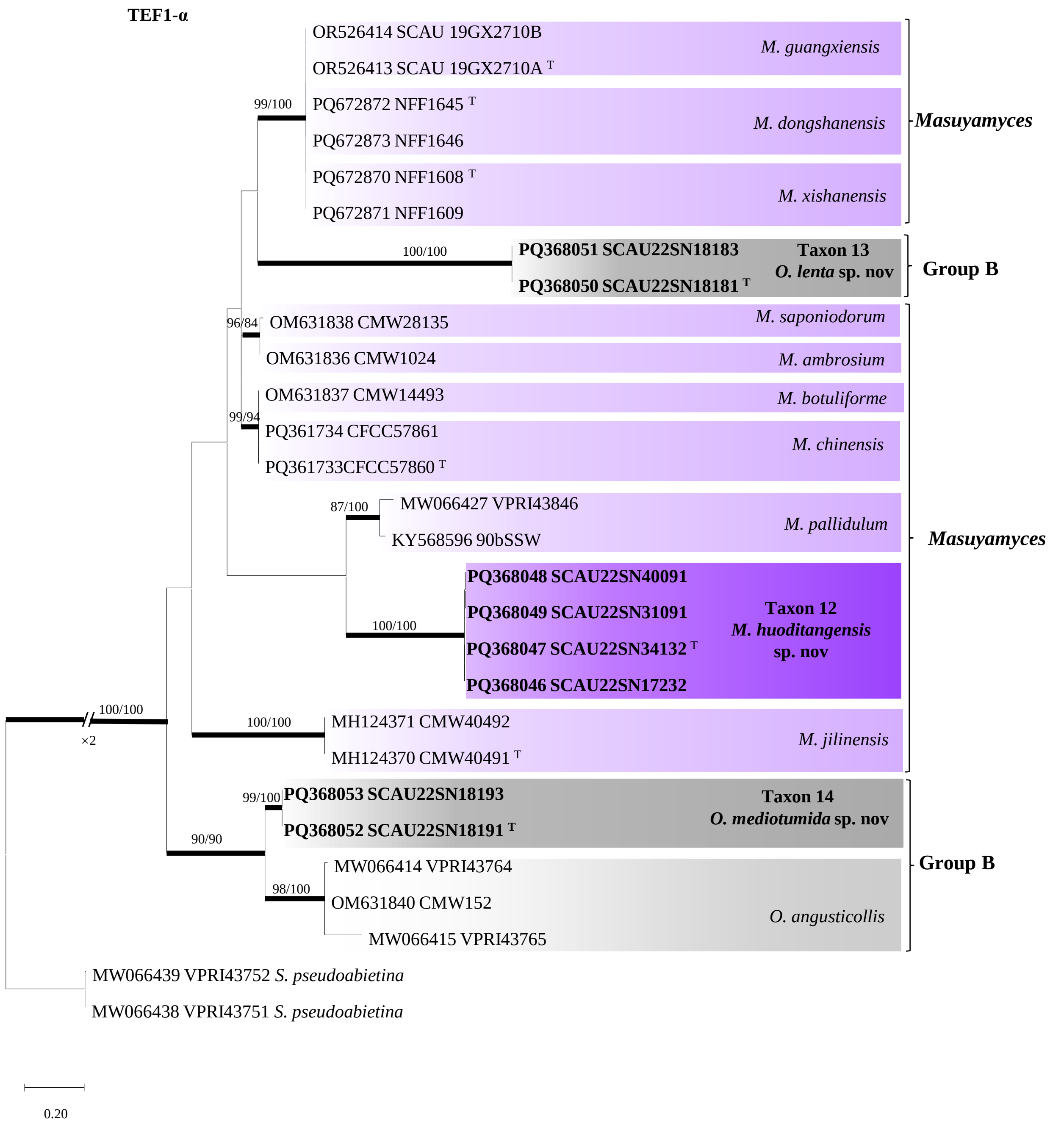

S5. Maximum Likelihood tree of *Masuyamyces* and Group B generated from the TEF1-α sequence data. Sequences generated from this study are printed in bold. Bold branches indicate posterior probability values ≥ 0.95. Bootstrap values of ML/MP ≥ 70% are recorded at the nodes. T = ex-type isolates.

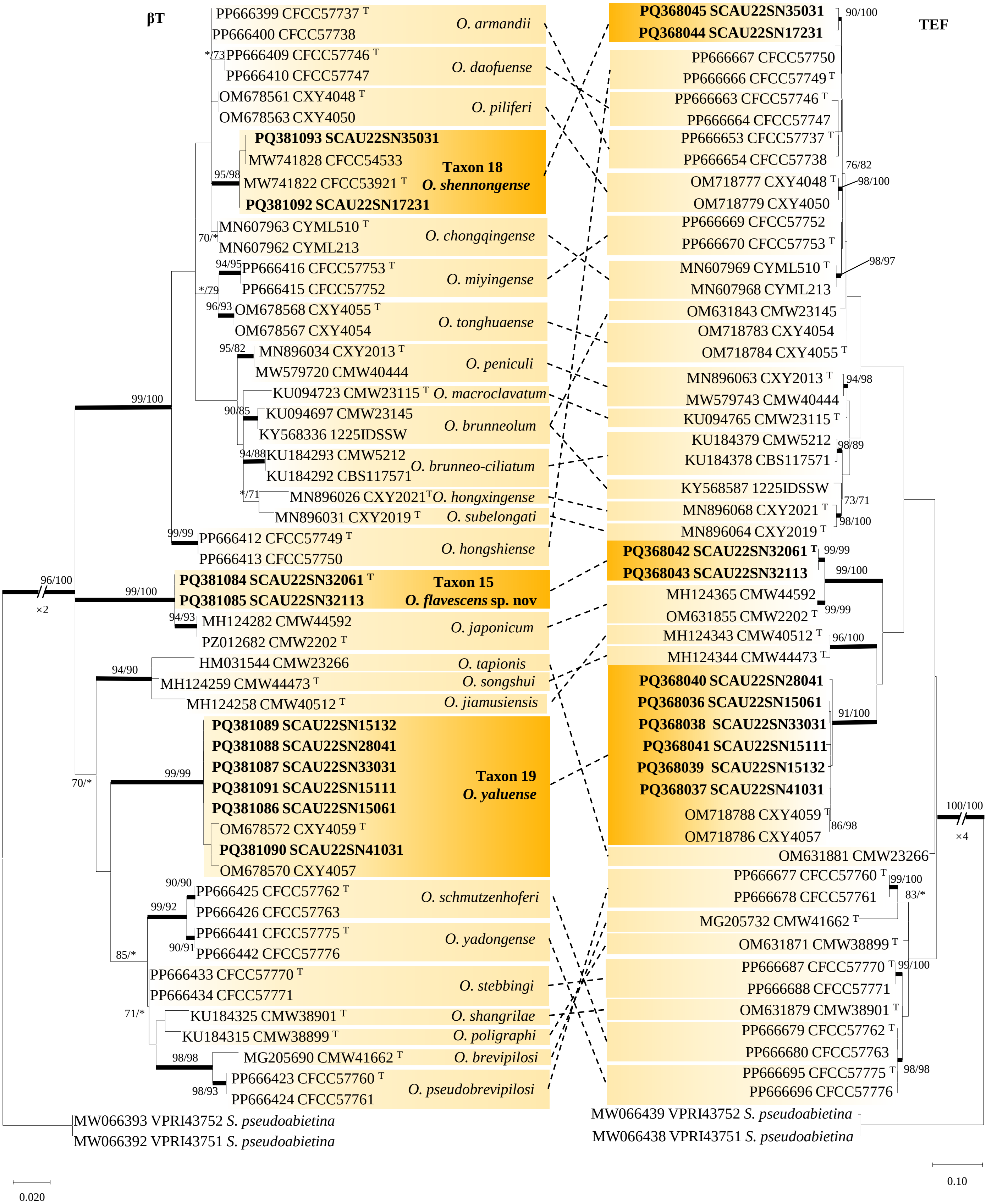

S6. Maximum Likelihood tree of *O. clavatum* complex generated from the βT and TEF1-α sequence data. Sequences generated from this study are printed in bold. Bold branches indicate posterior probability values ≥ 0.95. Bootstrap values of ML/MP ≥ 70% are recorded at the nodes. T = ex-type isolates.
